# Class Ib MHC–Mediated Immune Interactions Play a Critical Role in Maintaining Mucosal Homeostasis in the Mammalian Large Intestine

**DOI:** 10.4049/immunohorizons.2100090

**Published:** 2021-12-15

**Authors:** Suryasarathi Dasgupta, Igor Maricic, Jay Tang, Stephen Wandro, Kelly Weldon, Carolina S. Carpenter, Lars Eckmann, Jesus Rivera-Nieves, William Sandborn, Rob Knight, Peter Dorrestein, Austin D. Swafford, Vipin Kumar

**Affiliations:** * Division of Gastroenterology, Department of Medicine, University of California San Diego, La Jolla, CA; † Department of Biomedical Sciences, Cedars-Sinai Medical Center, Los Angeles, CA; ‡ Center for Microbiome Innovation, University of California San Diego, La Jolla, CA; § Collaborative Mass Spectrometry Innovation Center, Skaggs School of Pharmacy and Pharmaceutical Sciences, University of California San Diego, La Jolla, CA; ¶ Department of Pediatrics, School of Medicine, University of California San Diego, La Jolla, CA; ‖ Department of Computer Science and Engineering, University of California San Diego, La Jolla, CA; # Department of Bioengineering, University of California San Diego, La Jolla, CA

## Abstract

Lymphocytes within the intestinal epithelial layer (IEL) in mammals have unique composition compared with their counterparts in the lamina propria. Little is known about the role of some of the key colonic IEL subsets, such as TCRαβ^+^CD8^+^ T cells, in inflammation. We have recently described liver-enriched innate-like TCRαβ^+^CD8αα regulatory T cells, partly controlled by the non-classical MHC molecule, Qa-1^b^, that upon adoptive transfer protect from T cell–induced colitis. In this study, we found that TCRαβ^+^CD8αα T cells are reduced among the colonic IEL during inflammation, and that their activation with an agonistic peptide leads to significant Qa-1^b^–dependent protection in an acute model of colitis. Cellular expression of Qa-1^b^ during inflammation and corresponding dependency in peptide-mediated protection suggest that Batf3-dependent CD103^+^CD11b^−^ type 1 conventional dendritic cells control the protective function of TCRαβ^+^CD8αα T cells in the colonic epithelium. In the colitis model, expression of the potential barrier-protective gene, Muc2, is enhanced upon administration of a Qa-1b agonistic peptide. Notably, in steady state, the mucin metabolizing *Akkermansia muciniphila* was found in significantly lower abundance amid a dramatic change in overall microbiome and metabolome, increased IL-6 in explant culture, and enhanced sensitivity to dextran sulfate sodium in Qa-1b deficiency. Finally, in patients with inflammatory bowel disease, we found upregulation of HLA-E, a Qa-1^b^ analog with inflammation and biologic non-response, in silico, suggesting the importance of this regulatory mechanism across species.

## INTRODUCTION

Immune cells have a distinct pattern of distribution across the mammalian intestine both longitudinally and transversely, influenced at least in part by the microenvironmental niches in different anatomic locations (1). Thus, intestinal epithelial layers (IELs), which are lymphocytes localized in the epithelial barrier in the colon, may be part of the initial cellular defense that the host mounts to various insults from the luminal side and help decide the fate of the tissue resulting in homeostasis or pathology (2, 3). Comparatively little is known of colonic IELs compared with their counterparts in the underlying lamina propria (LP) tissue during inflammation (4). Recent single-cell RNA sequencing (scRNA-seq) studies in human colon have indicated the specialized environment in the epithelial layer where immune cells reside (5). In addition, how the microbiome, as another component of the gut epithelial microenvironment, helps shape this immune cell mediation in barrier function is poorly understood.

TCRαβ^+^CD8^+^ T cells are a major component of gut epithelial barrier community in both mice and humans (2, 3). They are of at least two varieties based on the CD8 dimers: innate-like (also named unconventional or natural) CD8^+^ T cells bearing CD8αα homodimers, and adaptive (conventional or inducible) CD8^+^ T cells carrying CD8αβ heterodimers (2–4). It is noteworthy that CD8αα is expressed by four different cell types in the IEL: TCRαβ^+^CD8αα^+^CD8αβ^+^, TCRαβ^+^CD8αα^+^ CD8αβ^−^, TCRγδ^+^CD8αα^+^, and TCR^−^CD8αα^+^. Because IELs are enriched among intestinal cells expressing CD8αα homodimers (2), we hypothesized that a careful analysis of TCRαβ^+^ CD8αα^+^ T cells may provide a biomarker for epithelial barrier function, a quintessential element that is compromised in inflammatory bowel disease (IBD) and its murine models. Although in human intestine, the presence of CD8αα^+^ T cells has been controversial (2, 6), recent evidence from scRNA-seq profiling reveals the TYROBP^+^ CD3^+^CD8^+^ IEL population as natural and the TYROBP^−^ CD3^+^CD8^+^ IEL population as the inducible counterpart to the murine cells in human colon (7, 8). Although both of these cell types were enriched in NK cell markers, the TYROBP^+^ population was reduced during inflammation whereas there was an expansion in the TYROBP^−^ population (8). The latter behavior suggests a homeostatic role for the TYROBP^+^CD8^+^ T IELs. Functionally, these innate-like CD8^+^ T cells have been mostly studied in the context of the small intestinal epithelial layer in mice. We have recently demonstrated innate-like TCRαβ^+^CD8αα^+^ regulatory T cells (Tregs) that, upon adoptive transfer, protect recipient mice from T cell–induced colitis in a perforin-dependent manner (9). These cells have a polyclonal TCR repertoire and an activated/memory phenotype and are distinct from NKT and mucosa-associated invariant T cells and, accordingly, are present in CD1d^−/−^ and Mr1^−/−^ mice as well as in germ-free mice. Additionally, these TCRαβ^+^ CD8αα^+^ T cells are Foxp3 negative, and a sizeable portion of them are restricted by the non-classical MHC molecule Qa-1b (9, 10). Recently, Qa-1b has been demonstrated to be a high-affinity functional ligand for the CD8αα homodimer (11). In addition, Qa-1b-restricted CD8^+^ T cells have previously been reported to be immunoregulatory T cells in experimental autoimmune encephalomyelitis (EAE) and lupus (12–15). The Qa-1-restricted TCRαβ^+^CD8^+^ T cells express CD44, CD122, and the NK inhibitory receptor Ly49CIFH (16–18). In addition to PLZF, innate-like regulatory TCRαβ^+^CD8^+^ T cells also express the transcription factor Helios (9, 19). How these innate-like T cells coordinate function, especially in the context of inflammatory assault in the much thicker mucus layer and a higher load of commensal microbiota adjacent to the colonic epithelium, is poorly understood. In this study, we demonstrate a unique immunoregulatory niche in the intestinal epithelial barrier in the steady state and in inflammation that can be specifically manipulated by engaging class Ib MHC-dependent TCRαβ^+^CD8αα^+^ T cell-type 1 conventional dendritic cell (cDC1)-mediated immune-microbiome interactions, to maintain mucosal homeostasis in colon. Additionally, using in silico methods and IBD datasets, we also probe human relevance for this unique pathway.

## MATERIALS AND METHODS

### Mice

C57BL/6 (B6) mice and Batf3^−/−^ mice on a B6 background were purchased from The Jackson Laboratory (Bar Harbor, ME). Qa-1^b−/−^ mice (originally provided by Dr. P. Jensen, University of Utah) on a B6 background were bred in our own facility in the University of California San Diego. For the microbiome comparison studies, age-matched wild-type (WT) B6 mice of different sexes were purchased and maintained for >6 mo, and differently aged mice feces were collected separately and compared. For colitis experiments, mice were kept in our animal house in the same room for at least 2 wk before experimentation.

Animal studies were carried out in strict accordance with the recommendations of the *Guide for the Care and Use of Laboratory Animals* of the National Institutes of Health. The protocols were reviewed and approved by the Institutional Animal Care and Use Committee of the University of California San Diego.

### Isolation of colonic cells

Colonic cell isolation was performed as previously published with a few modifications (20). Briefly, colons were removed, cleaned of mesentery and feces, and opened up longitudinally. They were then transversely cut into small pieces of ~1 cm in length, and intraepithelial and LP fractions were extracted following the manufacturer’s protocol (LP dissociation kit, a MACSmix tube rotator, Miltenyi Biotec). Following two rounds of predigestion solution using the MACSmix tube rotator, the IEL fraction was generated. The remaining tissue was washed to remove the EDTA, and supernatant was again pooled with the IEL fraction (third wash). The colon pieces were then incubated in digestion solution (containing enzymes) at 37°C and gently shaken as mentioned using the MACSmix tube rotator. Finally, tissue pieces were disrupted using the m_intestine_01 program of the gentleMACS dissociator and passed first through a 100-μm filter and then through a 70-μm filter to obtain the LP fraction.

For the small intestinal IEL fraction, a similar method was followed. For mesenteric lymph node (MLN), lymph nodes were carefully isolated and made free of extra fat and then physically mashed gently using the plunger of a 5-ml syringe while passing through a 70-μm filter to obtain single-cell suspensions.

### Flow cytometry

Single-cell suspensions from different tissue sources were first incubated with anti-CD16/CD32 (Fc block) in FACS buffer (0.02% sodium azide/2% FBS/PBS) and then surface stained with Abs against CD45 (clone 30-F11), CD45R/B220 (clone RA3–6B2), CD4 (GK1.5 or RM4–5), TCRb (H57–597), CD8a (53–6.7), CD8b (YTS156.7.7), CD8b0.2 (53–5.8), CD25 (7D4 or PC61), CD122 (TM-b1), Ly49CIFH (14B11), and CD44 (IM7) and subsequently stained intracellularly for Foxp3 (3G3) and Helios (22F6) for the T cell panel. Separate surface labeling was performed on the same single-cell suspension samples for the myeloid cell panel with Abs against CD45 (clone 30-F11), MHC class II (M5/114), CD11c (HL3 or N418), CD11b (M1/70), CD103 (2E7), CD3 (17A2 or 145–2C11), CD19 (6D5), Siglec H (551), PDCA-1 (129C1), CD45R/B220 (clone RA3–6B2), and Qa-1b (6A8.6F10.1A6). Abs were purchased from BD Biosciences, BioLegend, or Thermo Fisher Scientific. Following surface staining for each sample, live/dead staining was performed using eFluor 780 fixable viability live/dead dye staining (eBioscience), following the manufacturer’s protocol. Intracellular transcription factor staining was performed using Foxp3/transcription factor staining buffer set (eBioscience) according to manufacturer’s protocol.

Samples were further resuspended in BD Biosciences stabilizing fixative and stored at 4°C until acquisition. Cells were analyzed on a FACSCalibur or FACSCanto (BD Biosciences) at the Flow Cytometry Research Core Facility (Veterans Affairs San Diego Healthcare System, San Diego, CA), and all analyses were performed using FlowJo v10 software (Tree Star).

### Acute DSS-induced colitis

Mice were supplied with 2.5% or 1.5% DSS (molecular mass 40–50 kDa, Affymetrix, USB) in drinking water for 7 d with replenishment on day 3. After 7 d, DSS containing water was replaced with regular drinking water and mice were sacrificed at the peak of disease or at different time points as indicated. Body weight and water consumption were monitored regularly and mice were sacrificed by CO_2_ euthanasia followed by cervical dislocation. Mice were immunized i.p. (50 μg/mouse) with either a Qa-1b–binding 9-mer peptide LFFVLSSLL, which activates regulatory CD8^+^ T cells in B6 mice (H. Sheng, I. Marrero, I. Maricic, and V. Kumar, manuscript in preparation), emulsified in IFA or buffer in the control group once 4 d before starting DSS water.

### Histology and gross parameters in colitis model

After sacrifice, colons were isolated and cleaned. Cleaned colons were measured for length and stool softness. Colon length change indicates a decrease in colon length in centimeters from mean length (8 cm for male and 7.5 cm for female) of untreated B6 colons of matched age, sex, and similar housing. Stool softness was scored on a scale of 0–3: 0, normal; 1, mildly soft stool; 2, soft but not runny stool; 3, completely runny and bloody stool. The entire mouse colon barring the last 1 cm was either used for FACS or rolled up into “Swiss rolls” and fixed in Bouin’s fixative (RICCA Chemical, Arlington, TX). The fixed colon rolls were then sectioned and stained with H&E at the University of California San Diego Histology Core. The processed slides were scored in a blinded fashion by a trained pathologist for ulcerations in micrometers and then converted to centimeters. Finally, the colon length decrease in centimeters, stool softness score, and histological ulcer score in centimeters were summed to arrive at the cumulative colitis score on a scale of 0–20. Exceptions were made for Fig. 3E and Supplemental Fig. 2B, where histology scoring was not available due to utilization of tissue for a different purpose, and, accordingly, for these figures the *y*-axis was altered to reflect this change.

### Colon explant culture and analysis of supernatant

The last 1 cm of colon from the distal end was cut and washed twice in PBS. The washed piece of colon was then put in culture overnight in a 24-well plate with 1 μl of RPMI 1640 without serum but containing 1% penicillin and streptomycin. Following incubation, culture fluid was carefully collected and centrifuged, and supernatant was stored at −80°C for analysis later. ELISA or a cytometric bead array (CBA) kit was used to measure cytokines in the supernatant.

### Quantitative real-time PCR of murine genes

Total RNA was isolated from proximal and distal colon tissues using an RNeasy mini kit (Qiagen, 74104). RNA was quantified using a NanoDrop 2000c spectrophotometer (Thermo Fisher Scientific) and reverse transcribed using a Maxima first-strand cDNA synthesis kit for quantitative real-time PCR (Thermo Fisher Scientific, K1672). Real-time PCR was performed using Maxima SYBR Green/ROX quantitative PCR (qPCR) master mix (2×) (Thermo Fisher Scientific, K0241) on a StepOnePlus real-time PCR system (Applied Biosystems) with specific PCR primer pairs. Data were analyzed using relative mRNA gene expression over mRNA housekeeping gene (L32) expression.

### CBA assay

To measure IL-6, TNF-α, IFN-γ, and IL-17 released in supernatant from colon explant culture, a CBA mouse Th1/Th2/Th17 cytokine kit (BD Biosciences, 560485) was used. A CBA assay was performed according to the manufacturer’s protocol. Data were analyzed using FCAP Array (Soft Flow).

### 16S rRNA gene sequencing data acquisition and processing

Samples were processed and for 16S sequencing as previously described (21, 22). Briefly, the V4 hypervariable region of the 16S rRNA gene was amplified using barcoded 515f-806r primers and followed by 2 × 150-bp sequencing on the Illumina MiSeq. Raw sequences were processed in QIIME 2 within Qiita and denoised to sequence variants with deblur (23–25). α Diversity was calculated using the Shannon index after rarefaction to 6300 sequences per sample. β Diversity, measured by robust Aitchison distance, was calculated and a biplot was created using DEICODE (26). Statistical significance of difference between bacterial composition of genotypes was determined by permutational ANOVA (PERMANOVA) using the robust Aitchison distance matrix. The data have now been submitted to the European Nuclear Archive under accession number ERP132208 (http://www.ebi.ac.uk/ena/data/view/ERP132208).

### *Akkermansia* qPCR

For the quantitative detection of *Akkermansia muciniphila* DNA via qPCR, we used the following primers 5′-CAGCACGT-GAAGGTGGGGAC-3′ (forward) and 5′-CCTTGCGGTTGGCTT CAGAT-3′ (reverse) as described previously (27). Each amplification reaction was carried out in a total volume of 10 μl with 5 μl of SsoAdvanced universal SYBR Green supermix (Bio-Rad Laboratories), 0.5 μl of each primer (10 μM stock; Integrated DNA Technologies), 3.5 μl of PCR-grade water (Invitrogen), and 1 μl of purified genomic DNA. Standard curves for qPCR data analysis were generated based on 10-fold serial dilutions (from 5 to 0.0005 ng/μl) of cultured *A. muciniphila*. We then estimated gene copy counts by converting the detected DNA concentration by qPCR using the following equation: Gene copies = (DNA concentration (nanograms) × Avogadro’s number/amplicon length (base pairs) × Daltons) × 1.0E+09, where amplicon length was determined using National Center for Biotechnology Information reference sequence NR_042817.1.

### Metabolomics data acquisition and processing

Mass spectrometric data were generated and processed using a previously published protocol (28). In brief, samples were lyophilized, resuspended in 50% MeOH 50% water (Optima liquid chromatography–mass spectrometry grade; Fisher Scientific, Fair Lawn, NJ), and analyzed on an ultra-HPLC system (UltiMate 3000; Thermo Fisher Scientific, Waltham, MA) coupled to a Maxis quadruple time of flight mass spectrometer (Bruker Daltonics, Bremen, Germany) with a Kinetex C18 column (Phenomenex, Torrance, CA) in the positive electrospray ionization mode using a linear gradient of the mobile phase of water with 0.1% formic acid (v/v) and acetonitrile with 0.1% formic acid (v/v) (liquid chromatography–mass spectrometry-grade solvents; Fisher Chemical). MZmine2 (version MZmine-2.37.corr16.4) (18) was used for MS1 feature finding as previously described (28), followed by feature-based molecular networking using GNPS (29). The feature table was normalized by converting each sample to relative abundances. β Diversity was calculated and biplot was created using DEICODE. Statistical significance of difference between metabolome composition of genotypes was determined by PERMANOVA using the robust Aitchison distance matrix. Log ratios of bile acids were calculated by summing intensities of bile acid subtypes within each sample, followed by taking the log ratio of primary-to-primary–conjugated bile acids within each sample.

### Bioinformatics analysis of human datasets

Human gene expression data were downloaded from the Gene Expression Omnibus database (accession numbers GSE16879 [https://www.ncbi.nlm.nih.gov/geo/query/acc.cgi?acc=GSE16879], GSE73661 [https://www.ncbi.nlm.nih.gov/geo/query/acc.cgi?acc=GSE73661], and GSE100833 [https://www.ncbi.nlm.nih.gov/geo/query/acc.cgi?acc=GSE100833]). The data were then background corrected, normalized with the robust multiarray averaging procedure, and transformed with an oligonucleotide package (30) from R with Bioconductor version 3.10. The gene expression difference was examined by two-way ANOVA in R. Similarly, human proteomics data were downloaded from the Proteomics Identification Database with accession number PXD012284 (https://www.ebi.ac.uk/pride/archive/projects/PXD012284). The data were variance stabilization–normalized and missing values were imputed with the DEP package (31) from R with Bioconductor version 3.10. Human scRNA data were downloaded from Single Cell Portal by accession number SCP259 (https://singlecell.broadinstitute.org/single_cell/study/SCP259/intra-and-inter-cellular-rewiring-of-the-human-colon-during-ulcerative-colitis), with cell type assignment from the original publication (5). The differential expression analysis was performed by the MAST generalized linear model framework (32) at default parameters with implementation in the Seurat package version 3.1.4 (33).

### Statistical analysis

Data were analyzed using GraphPad Prism v7 software (GraphPad Software). Data are reported as mean ± SEM. The two-tailed unpaired *t* test, Student *t* test, or Mann–Whitney *U* test was used when comparing two groups of unpaired data. A one-way ANOVA with the Bonferroni multiple comparison posttest was used when comparing three or more groups. Significance was assessed using two-tailed tests and is indicated as follows: *p < 0.05, **p < 0.01, ***p < 0.001, ****p < 0.0001.

## RESULTS

### TCRαβ^+^CD8^+^ T cell niche in colonic IEL is different from other mucosal compartments in steady state

**FIGURE 1.**
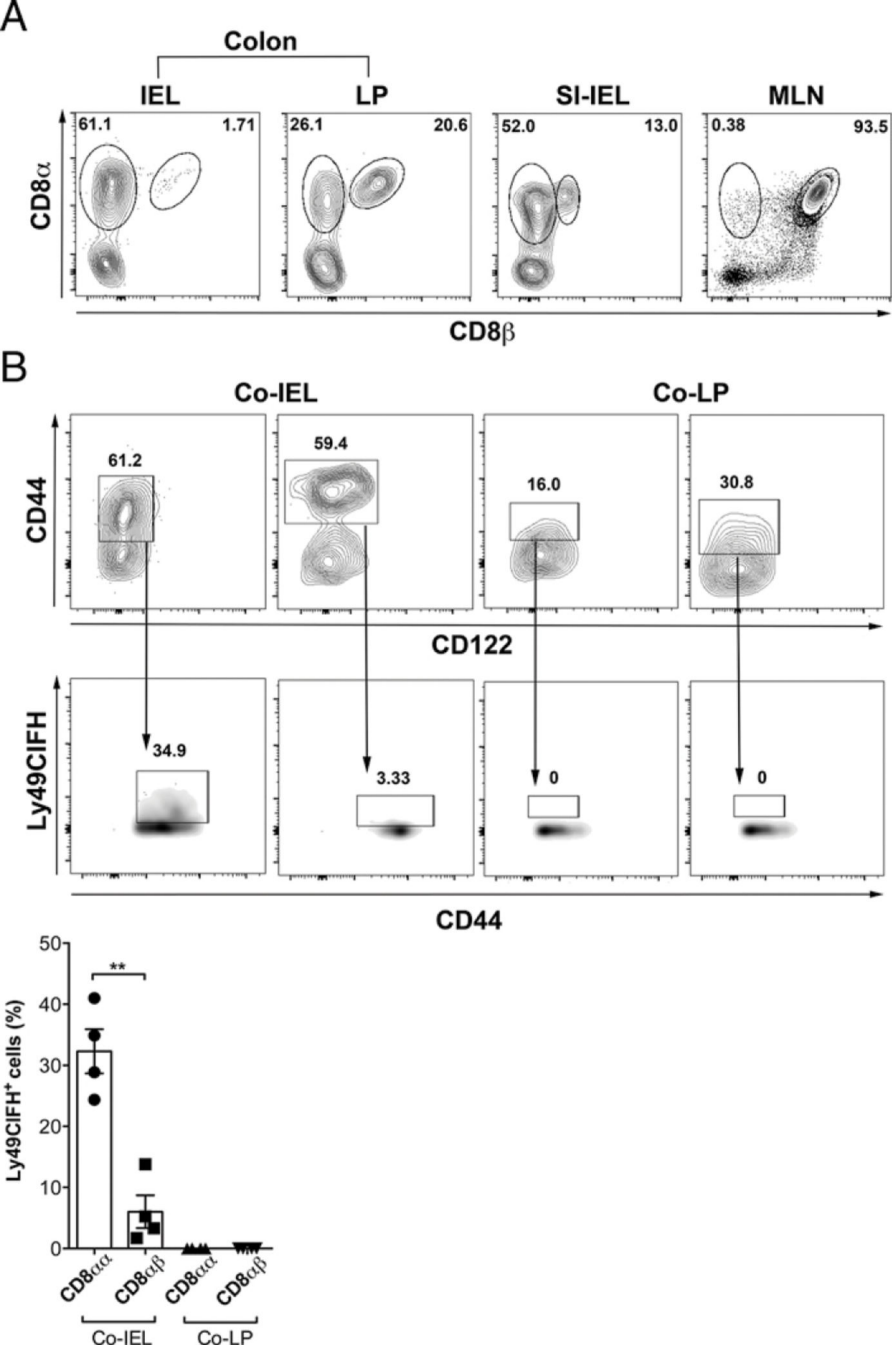
In steady state, colonic IEL harbors a rich proportion of TCRαβ^+^CD8αα^+^ T cell subsets **(A)** Representative images from three independent experiments, showing comparative frequency of TCRαβ^+^CD4^−^B220^−^CD8αα^+^ and TCRαβ^+^ CD4^−^B220^−^CD8αβ^+^ T cells in colonic IEL, colonic LP, small intestinal IEL, and MLN from WT B6 female mice (*n* = 4–5). Gates on each flow plot indicate percentage of CD8αα^+^ (left one) and CD8αβ^+^ (right one) T cells in each tissue. (**B**) CD8αα^+^ T cells in colonic IEL express NK inhibitory receptors. Gates in the contour plots (upper panels) indicate CD44^+^CD122^lo/−^ events in CD8αα^+^ (left panel) or CD8αβ^+^ (right panel) T cells for colonic IEL or colonic LP as indicated. Gates in the density plots (lower panels) indicate Ly49CIFH^+^ events within the selected gating. Bar graph represents quantification of percentage of Ly49CIFH^+^CD44^+^ CD122^lo/−^ cells in the four cell populations. Each dot in the bar graph represent one animal. Data are representative of two independent experiments. Error bars represent mean ± SEM. A student *t* test was used to determine statistical significance. Co-IEL, colonic IEL; Co-LP, colonic LP; SI-IEL, small intestinal IEL. **p < 0.01.

To gain a better understanding of steady-state composition of TCRαβ^+^CD8^+^ T cells, we first analyzed the presence of innate-like TCRαβ^+^CD4^−^B220^−^CD8αα^+^ T cells and their adaptive counterpart TCRαβ^+^CD4^−^B220^−^CD8αβ^+^ T cells in different gut mucosal compartments: colon IEL and LP fractions, small intestinal IEL, and MLN from naive B6 mice. We found that the frequency of TCRαβ^+^CD8αα^+^ T cells in colonic IEL (as compared with other compartments) was much higher than that of CD8αβ^+^ T cells (Fig. 1A). Next, we determined whether the cell surface phenotype of TCRαβ^+^CD8αα^+^ cells is similar to that displayed by regulatory CD8^+^ T cells. We found that the TCRαβ^+^CD8αα^+^ T cell population among the colon IEL expresses two markers, CD44 and the NK inhibitory receptors Ly49CIFH, whereas the expression of CD122 is low/no. In contrast, neither the TCRαβ^+^CD8αα T cells in colon LP nor CD8αβ^+^ T cells in colonic IEL or colon LP express Ly49CIFH (Fig 1B). Consistent with their regulatory phenotype, colonic TCRαβ^+^CD8αα^+^ T cells predominantly were Helios^+^ Foxp3^−^ in the colon IEL fraction in comparison with the colon LP cells (Supplemental Fig. 1A). Similar to the unique niche of CD8^+^ T cells in murine colonic IEL, in silico analysis of CD8^+^ cells in the epithelial layer versus LP in healthy human colon also revealed a differential gene expression profile underlining the uniqueness that intraepithelial residence provides to these lymphocytes in the steady state (Supplemental Fig. 1B). Our murine data indicate a unique phenotype of the colon IEL TCRαβ^+^CD8αα^+^ T cells containing potential regulatory CD8^+^ populations in the steady state in naive B6 mice.

### Colonic IEL TCRαβ^+^CD8αα^+^ and CD8αβ^+^ T cells correlate differentially with induction and restoration phases in an acute model of colitis

**FIGURE 2.**
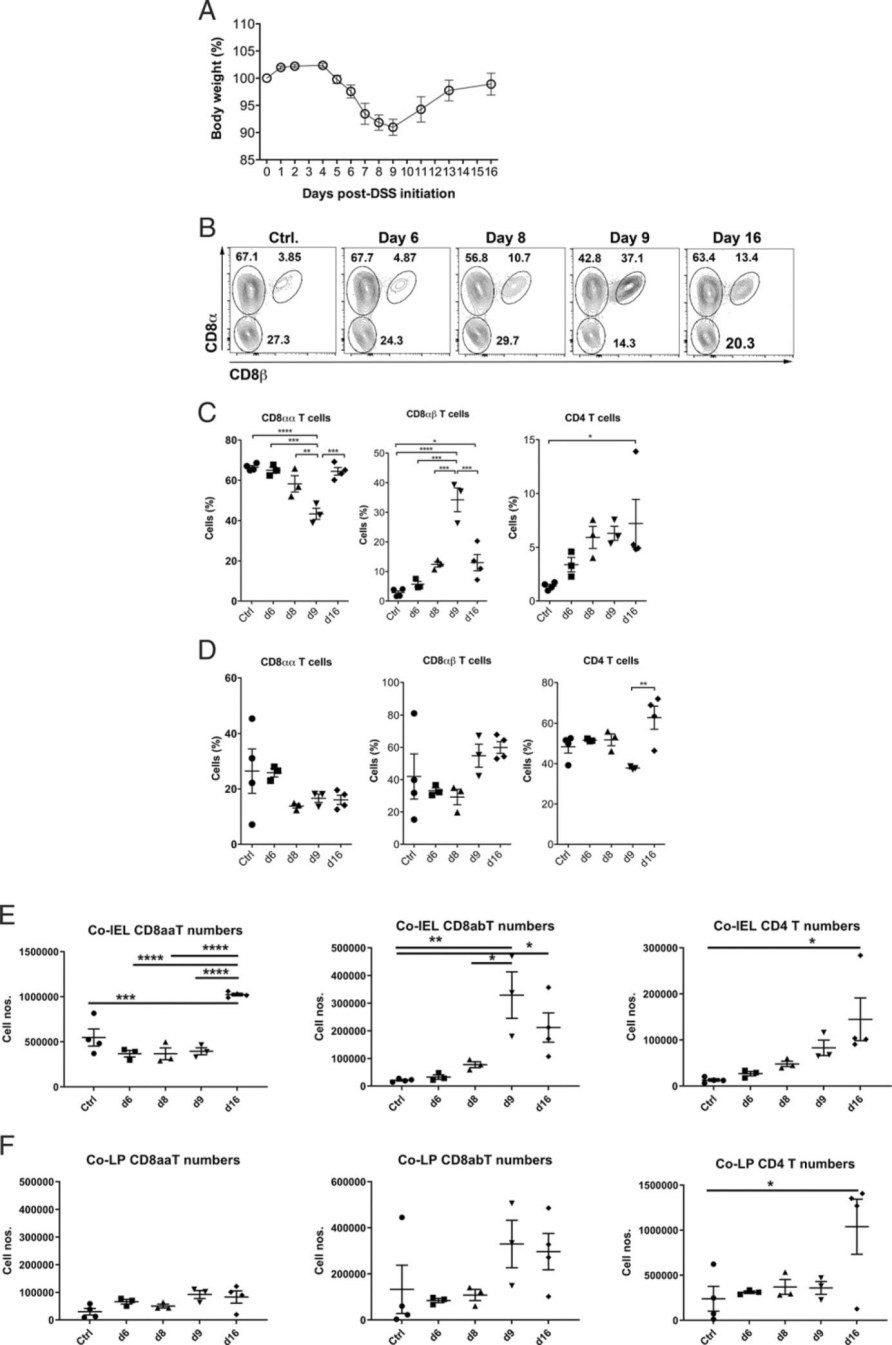
TCRαβ^+^CD8^+^ T cell subsets in colonic IEL are a marker of pathology and restitution in an acute model of colitis (**A**) Body weight loss curve in acute DSS colitis. WT B6 mice (*n* = 3–5 per group) were fed 2.5% DSS (or not for vehicle controls) in water for 7 d and then switched to regular water. (**B**) Representative Flow plots showing CD45^+^ TCRαβ^+^CD4^−^B220^−^ events on different days in the acute DSS colitis. CD8αα^+^ T cells (top left gate in each plot) correlate negatively with induction and positively with restoration phases in an acute model of colitis in contrast to CD8αβ^+^ (top right gate in each plot) T cells. (**C**) Scattered plots indicate percentage of CD8αα^+^ (left panel), CD8αβ^+^ (middle panel), and CD4^+^ (right panel) in CD45^+^ TCRαβ^+^CD4^−^B220^−^ T cells from colonic IEL. Each dot represents one animal. (**D**) Scattered plots indicate percentage of CD8αα^+^ (left panel), CD8αβ^+^ (middle panel), and CD4^+^ (right panel) in CD45^+^ TCRαβ^+^CD4^−^B220^−^ T cells from colonic LP. (**E** and **F**) Absolute cell numbers for T cell subsets shown in (C) and (D) calculated using live-gated events extrapolated by total cell numbers using the trypan blue method. Each dot represents one animal. Data are representative of three independent experiments. Error bars represent mean ± SEM. One-way ANOVA was used to determine statistical significance. Co-IEL, colonic IEL; Co-LP, colonic LP. *p < 0.05, **p < 0.01, ***p < 0.001, ****p < 0.0001.

Next, we determined the dynamics of different TCRαβ^+^CD8^+^ T cell subsets during acute inflammation in the colon. We used body weight loss as a general disease parameter in an acute model of DSS colitis. Mice started to lose body weight on day 5 after initiation of DSS (2.5%), with the peak of weight loss occurring on day 9 and subsequent weight gain (Fig. 2A). We found that the percentages of TCRαβ^+^CD8αα^+^ but not CD8αβ^+^ T cells in the colon IEL were markedly decreased during the peak disease phase (Fig. 2B, 2C). In contrast, their percentage in the LP was not significantly affected under these conditions. In comparison, TCRαβ^+^CD8αβ^+^ T cells increased by 10-fold among the colon IELs during the most severe phase of the disease, whereas CD4 T cells increased later in the recovery phase (Fig. 2C). In the LP fraction, we could only observe an increase in CD4^+^ T cells during the restoration phase (Fig. 2D). Thus, a significant increase in TCRαβ^+^CD8αβ^+^ T cells in the colon IEL during inflammation suggests that they might contribute to disease, whereas a significant decrease in TCRαβ^+^CD8αα^+^ T cells is consistent with a regulatory role. The quantification of absolute cell numbers also concurred with a significant accumulation of TCRαβ^+^CD8αβ^+^ T cells (Fig. 2E). Although the decrease in TCRαβ^+^CD8αα^+^ T cells was not significant, their increase was significant during the restoration phase (Fig. 2E). In addition, an enhancement in CD4 T cell numbers in IEL and LP was observed, but no significant alterations were found in CD8^+^ T cell subset numbers in LP (Fig. 2E, 2F).

### Administration of an agonistic peptide for the TCRαβ^+^CD8αα^+^ T cells protects colitis in WT mice but not in Qa-1b^−/−^ mice

**FIGURE 3.**
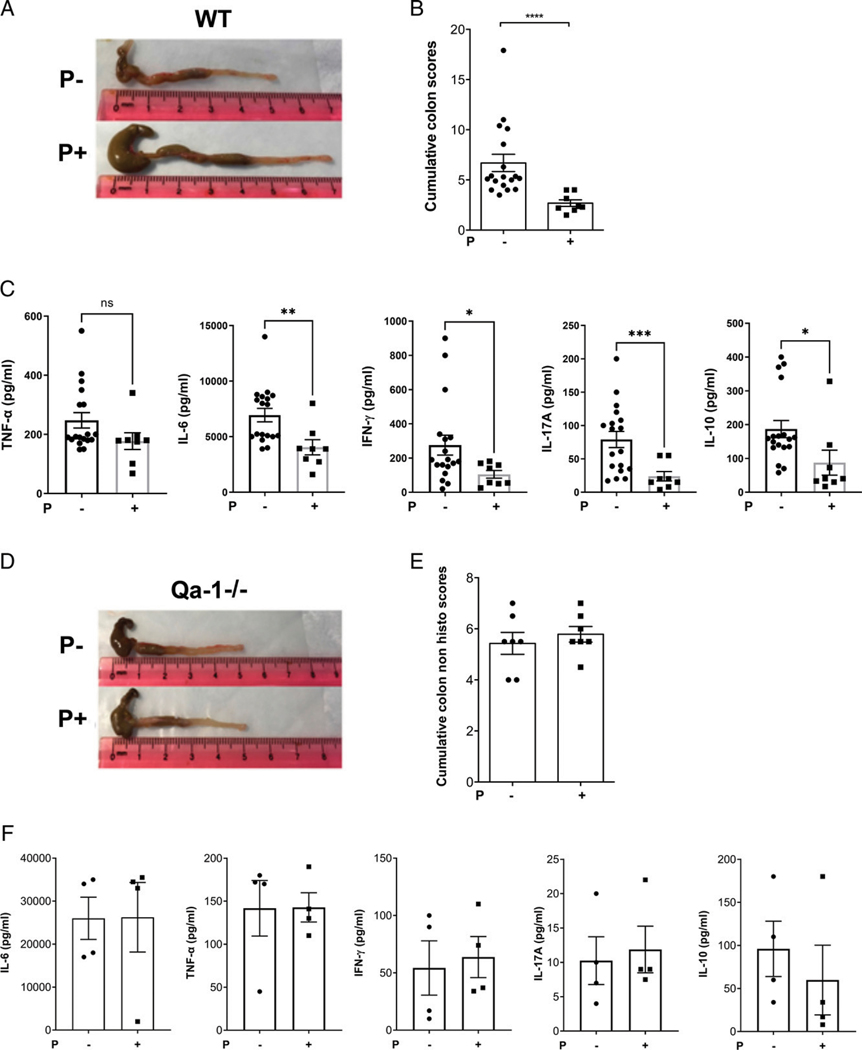
Peptide-mediated protection from colitis is Qa-1b–dependent (**A**) Representative photographs, from three independent experiments, showing colons from control-treated (P−, upper panel) or peptide-treated (P1, lower panel) WT B6 mice (*n* = 4–7) from acute DSS colitis model. (**B**) Bar graph showing cumulative colitis scores of control- or peptide-treated WT B6 mice from acute DSS colitis model. Each dot represents one animal. Error bars represent mean ± SEM. Non-parametric (Mann–Whitney) test was used to determine statistical significance. ****p < 0.0001. (**C**) Bar graphs showing concentration (pg/ml) values from CBA kit analysis of cytokines (TNF-α, IL-6, IFN-γ, IL-17A, and IL-10) released in supernatant from colon explant culture. Colons were from control- or peptide-treated WT B6 mice from acute DSS colitis model. A Student’s *t* test was used to determine statistical significance. *p < 0.05, **p < 0.01, ***p < 0.001. (**D**) Representative photographs showing colons from control-treated (P−, upper panel) or peptide-treated (P1, lower panel) Qa-1b^−/−^ mice from acute DSS colitis model. (**E**) Bar graph showing cumulative colitis scores (without histological scores) of control-or peptide-treated Qa-1b^−/−^ mice from acute DSS colitis model. (**F**) Bar graphs showing concentration (pg/ml) values from CBA kit analysis of cytokines (TNF-α, IL-6, IFN-γ, IL-17A, and IL-10) released in supernatant from colon explant culture. Colons were from control- or peptide-treated Qa-1b^−/−^ mice from acute DSS colitis model. Each dot represents one animal. Error bars represent mean ± SEM.

We have recently identified an agonistic 9-mer peptide that stimulates Qa-1 restricted regulatory TCRαβ^+^CD8αα^+^ T cells in vitro and in vivo and protects mice from EAE (H. Sheng, I. Marrero, I. Maricic, and V. Kumar, manuscript in preparation). Since TCRαβ^+^CD8αα^+^ T cells are decreased during colitis, we wanted to investigate whether prophylactic in vivo stimulation of these T cells with the agonistic peptide would confer protection in the acute model of colitis. Accordingly, we found that the administration of the agonistic peptide in WT B6 mice resulted in a significant reduction in gross colon parameters and cumulative colitis scores on the day of peak body weight loss (Fig. 3A, 3B). Explant cultures from peptide-treated mice also showed significant reduction in proinflammatory cytokine production (Fig. 3C). Although we observed that the peptide-treated mice gained weight after DSS treatment, we found that this weight gain was due to the IFA used to emulsify the peptide, and, therefore, in IFA-only controls, body weight was enhanced but without any effect on other colonic disease parameters (Supplemental Fig. 2A, 2B). To avoid this confounding variable, for further experiments with the peptide, we omitted body weight loss as a disease parameter and focused instead on other disease parameters to measure colitis. Next, to determine whether the peptide-mediated protection is Qa-1–dependent, we administered peptide in mice deficient in Qa-1b. In contrast to WT mice, peptide treatment failed to provide protection in Qa-1b^−/−^ mice as observed by gross colonic parameters and cytokines in explant cultures (Fig. 3D–F). These data indicate that the protection from colitis induced following peptide treatment is Qa-1^b^-dependent.

### CD103^+^CD11b^−^ cDCs populate colonic IEL during peak of DSS-induced colitis and uniquely upregulate Qa-1^b^

**FIGURE 4.**
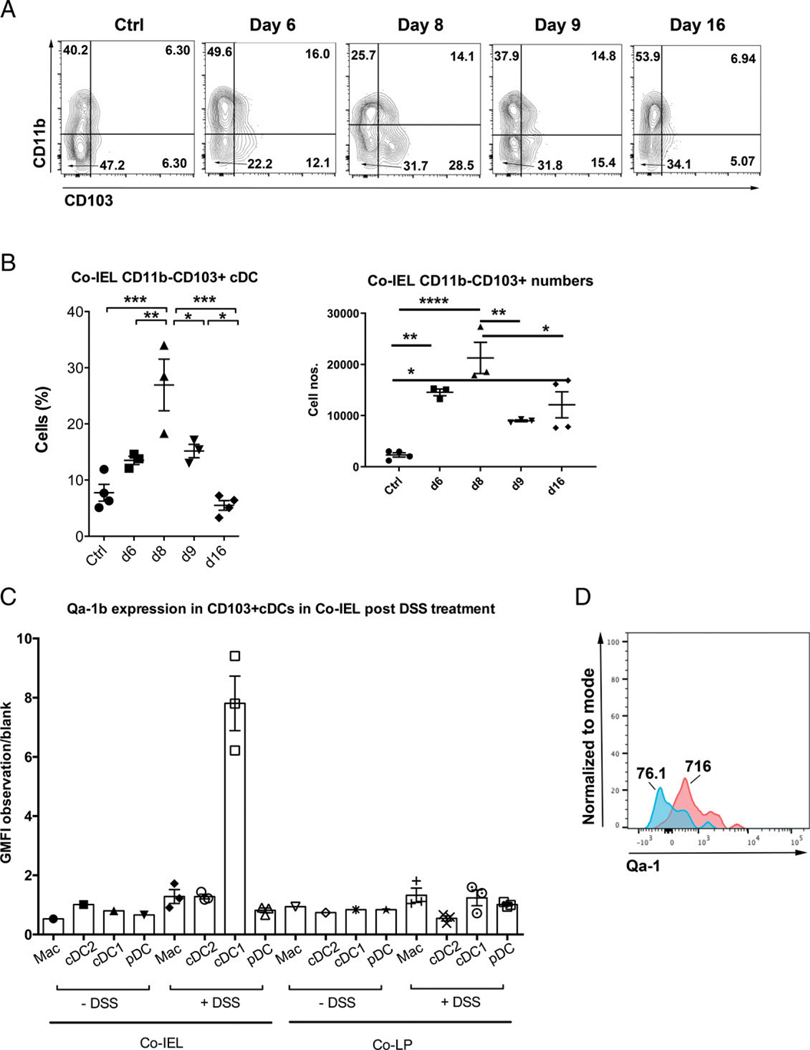
Batf3-dependent CD103^+^CD11b^−^ cDCs accumulate in colonic IEL and are enriched in Qa-1b expression (**A**) Representative flow plots from two independent experiments, showing CD45^+^MHC class II^+^CD3^−^CD19^−^CD11c^+^CD11b^−^CD103^+^ cDC events (bottom right quadrant) in colonic IEL on different days in acute DSS colitis in B6 mice (*n* = 3–5 per group). (**B**) Scattered plot showing percentage (left panel) of CD11b^−^CD103^+^ cDCs in CD45^+^MHC class II^1^CD3^−^CD19^−^CD11c^+^ cells and absolute numbers (right panel) in colonic IEL. One-way ANOVA was used to determine statistical significance. *p < 0.05, **p < 0.01, ***p < 0.001, ****p < 0.0001. (**C**) Bar graph showing ratio of geometric mean fluorescence intensity (GMFI) for Qa-1b expression over fluorescence minus one control for each myeloid cell population (macrophages [Macs] and DCs) in colon (IEL or LP) with or without inflammation on day 8 of acute DSS colitis model. (**D**) Representative histogram overlay for Qa-1b expression on CD103^+^CD11b^−^ cDCs over fluorescence; blue (MFI = 76.1) represents label control whereas pink (MFI = 716) represents labeled with anti–Qa-1 mAb. Each dot represents one animal. Error bars represent mean ± SEM. Co-IEL, colonic IEL; Co-LP, colonic LP.

We have previously demonstrated that DC-mediated Ag cross-presentation is required to induce TCRαβ^+^CD8αα^+^ T cells and facilitate their immunoregulatory function in EAE (34). There are several well-known subsets of professional APCs in mammalian intestine, and some of them have been implicated in immunoregulation (35–37). Of these subsets, cDC1s, characterized as CD103^+^CD11b^−^ in the murine intestine, are also known to display enhanced Ag cross-presentation properties in several situations (38, 39). Recently, intestinal cDC1s in mice have been reported to maintain tolerance to epithelial Ags involving a Foxp3^1^CD8^+^ Treg-inducing mechanism (40). As a first step toward determining whether DCs play a role in mediating the anti-inflammatory functions of CD8αα^+^ T cells, we characterized the phenotypes of DCs in the intraepithelial compartment, where TCRαβ^+^CD8αα^+^ T cells mainly reside, during colitis. We found a significant increase in the percentage of several DC subsets in the IEL fraction, including DCs that are barely present in the IEL fraction in the steady state, such as CD103^+^CD11b^−^ cDCs, but they were mostly found in the LP fraction. Additionally, DCs that we found to be present in the steady-state IEL included plasmacytoid DCs (pDCs) and CD103^−^CD11b^+^ cDCs (Fig. 4A, 4B, Supplemental Fig. 2C). Thus, we found that CD103^+^CD11b^−^ cDCs (cDC1s), but not pDCs or CD103^−^CD11b^+^ cDCs (cDC2s), significantly decreased, by frequency and numbers, among colon IEL after day 8, which correlated with the change in TCRαβ^+^CD8^+^ T cells at that time. Based on absolute numbers, we also found a general increase in both LP and IEL on day 16 compared with other days (Supplemental Fig. 2C). Next, we determined the expression of Qa-1b among various DC subsets and macrophages during colitis in colon IEL from WT mice. Analysis of the surface levels (geometric mean fluorescence intensity) of Qa-1b revealed that CD103^+^CD11b^−^ cDCs in colon IEL are the major Qa-1b-expressing DC population (Fig. 4C, 4D). Thus, a significant correlation between the numeric changes in Qa-1b^+^ CD103^+^CD11b^−^ cells and in the relevant subsets of TCRαβ^+^CD8^+^ T cells suggests that cDC1s may be important for the peptide-mediated Qa-1b-dependent protection from colitis.

### Peptide-mediated protection from DSS-induced colitis is lost in Batf3^−/−^ mice lacking cDC1s

**FIGURE 5.**
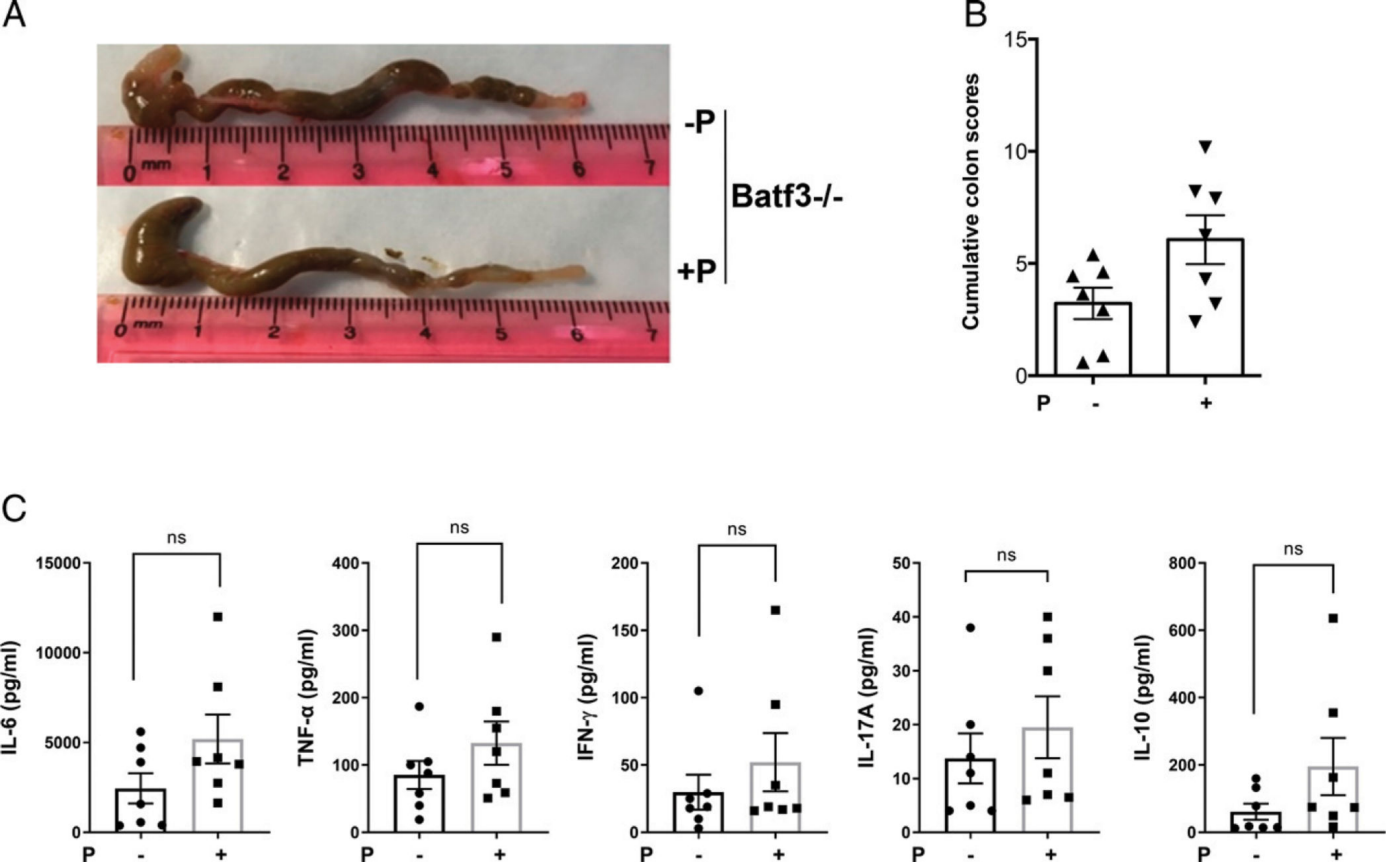
Role of Batf3-dependent cDCs in peptide-mediated protection from colitis (**A**) Representative photographs from two independent experiments showing colons from control-treated (−P, upper panel) or peptide-treated (+P, lower panel) Batf3^−/−^ mice (*n* = 4–7) from acute DSS colitis model (2.5%). (**B**) Bar graph showing cumulative colitis scores of control- or peptide-treated Batf3^−/−^ mice from acute DSS colitis model. (**C**) Bar graphs showing concentration (pg/ml) values from CBA kit analysis of cytokines (IL-6, TNF-α, IFN-γ, IL-17A, and IL-10) released in supernatant from colon explant culture. Colons were from control or peptide-treated WT B6 mice from acute DSS colitis model. Each dot represents one animal. Error bars represent mean ± SEM.

CD103^+^CD11b^−^ cDCs are known to be developmentally controlled by various transcription factors, including Batf3 (41). Batf3 also controls CD8αα^+^ cDCs in the spleen and other lymphoid tissues that, along with CD103^+^ cDCs form the cDC1s, are known for playing a role in intestinal antiviral CD8^+^ T cell-mediated immunity and tolerance induction (42–45). We first confirmed that Batf3^−/−^ mice have a substantially reduced number of CD103^+^CD11b^−^ cDCs in the colon (Supplemental Fig. 3A). Additionally, consistent with a compromised CD8^+^ T cell function in Batf3^−/−^ mice (46), the frequency of TCRαβ^+^CD8αβ^+^ T cells was also reduced in both colonic IEL and LP in these mice (Supplemental Fig. 3B). Importantly, we found that the protection induced by the Qa-1 agonistic peptide against acute colitis (with 2.5% DSS) was lost in Batf3^−/−^ mice in comparison with WT mice (Fig. 5). Importantly, however, note that the severity of colitis observed in Batf3^−/−^ mice was apparently lower in comparison with that in the WT mice. To address this potential confounding factor, we induced colitis in Batf3^−/−^ mice with an increased dose of DSS (3.5%), but again observed that peptide-mediated protection was lost in these mice (Supplemental Fig. 3C). These data suggest that the immune regulation of colitis is dependent on the presence of the Batf3-dependent Qa-1b^+^ cDC1 population in the inflamed epithelial layer of the colon.

### Peptide-mediated protection results in a selected upregulation of barrier function genes including Muc2 in distal but not in proximal colonic tissue

**FIGURE 6.**
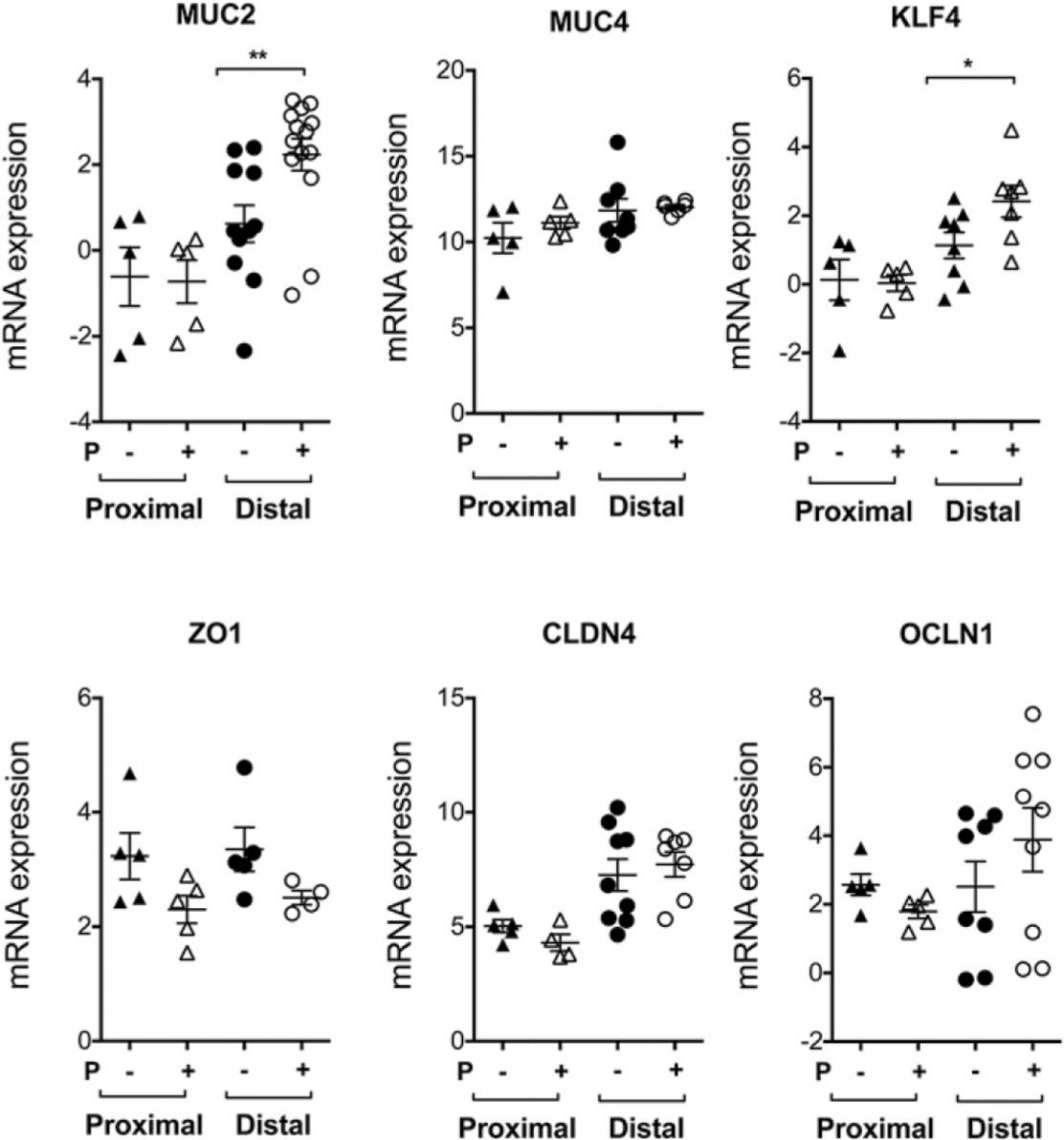
Administration of a Qa-1b agonist peptide increases expression of certain genes in distal colon known for barrier function enhancement Scattered plots showing relative mRNA expression (over housekeeping gene, L32) in proximal and distal colon from control- or peptide-treated animals in the acute DSS colitis model in WT B6 mice (*n* = 5–9). Each dot represents one animal. Data are representative of two independent experiments. Error bars represent mean ± SEM. A Student’s *t* test was used to determine statistical significance. *p < 0.05, **p < 0.01.

We wanted to determine whether the CD8^+^ T cell/CD103^+^ DC–mediated immune regulation impacts epithelial functions. We used qRT-PCR analysis to investigate changes in the expression of colon-expressed genes known for barrier protection in peptide-treated mice in comparison with the control group following DSS-induced colitis. We analyzed proximal and distal colon separately since they have displayed differential gene expression (47, 48) and observed that only in the distal, but not proximal, colon peptide administration enhanced the barrier function gene, Muc2 (Fig 6). Notably, Muc2 deficiency causes spontaneous colitis in mice (49). Muc2 has been shown to induce tolerogenic DCs in mice and humans and maintains gut homeostasis and oral tolerance (50). The other important gene found to be upregulated in the distal colon was Krüppel-like factor 4 (Klf4), and there were nonsignificant trends observed in the junctional molecule occludin (Ocln), but there were no trends observed in other molecules investigated, that is, MUC4, ZO1, and CLDN4 (Fig. 6). These data suggest that peptide-mediated activation of TCRαβ^+^CD8αα^+^ T cells in colon enhances expression of genes whose products play a key role in the gut barrier function.

### In the steady state, lack of Qa-1^b^ is associated with a dramatically altered microbiome and metabolome

**FIGURE 7.**
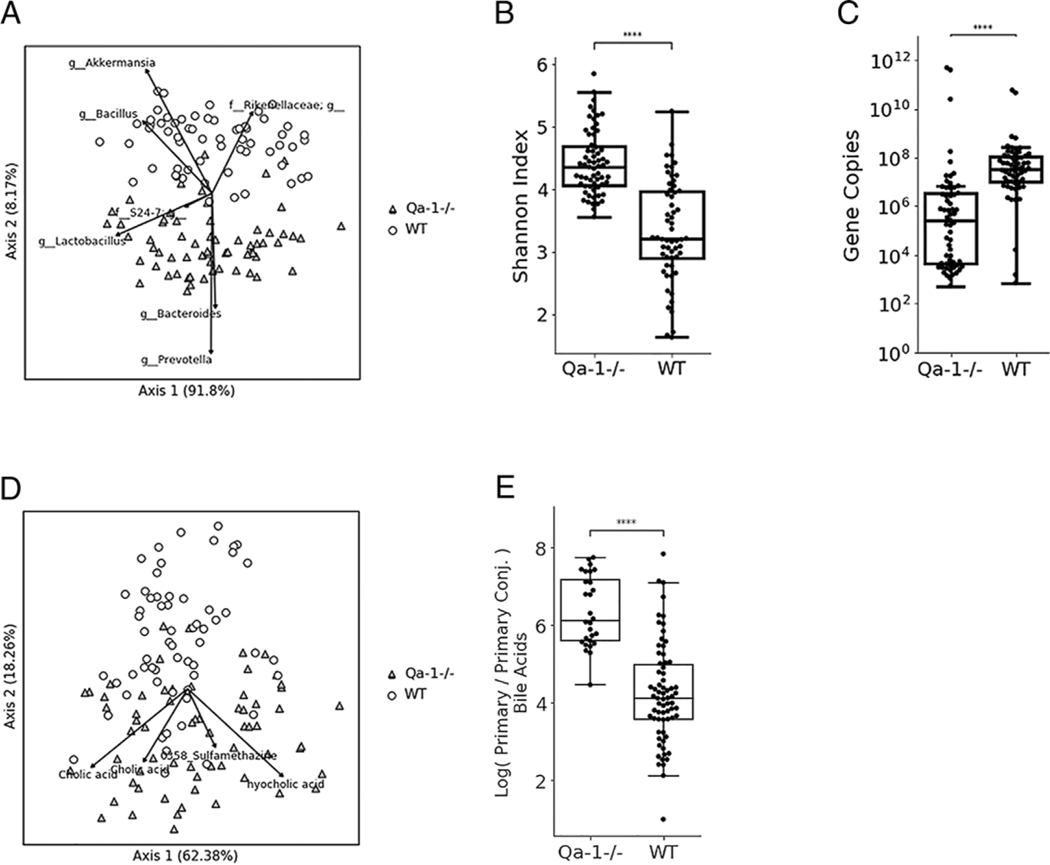
Alteration of stool microbiome and metabolome in absence of Qa-1b (**A**) Biplot of PCA using robust Aitchison distance of the fecal microbiota using the Earth Microbiome Project protocol 16S V4 amplicon sequencing to assess b diversity between samples and groups. The bacterial composition of Qa-1^−/−^ mice (gray triangles) is significantly different from WT mice (open circles) (PERMANOVA *F* = 73.5, *p* < 0.001). Arrows show the bacterial genera that contribute most to separation on these axes. (**B**) α Diversity of fecal microbiota measured by Shannon index (Mann–Whitney *U* = 3166, *p* = 3.8e−12). (**C**) Abundance of *Akkermansia* marker gene copies measured by qPCR (Mann–Whitney *U* = 564, *p* = 2.8e−11). (**D**) Biplot of PCA using robust Aitchison distance of the fecal metabolome based on untargeted liquid chromatography–tandem mass spectrometry. The metabolite composition of Qa-1^−/−^ mice (gray triangles) is significantly different from WT mice (open circles) (PERMANOVA *F* = 14.5, *p* < 0.001). Arrows show identified metabolites that contribute most to separation on these axes. (**E**) Qa-1^−/−^ mice had a higher ratio of primary bile acids to primary conjugated bile acids compared with WT mice based on fecal metabolomics (Mann–Whitney *U* = 178, *p* = 3.6e−10). Each symbol represents one individual mouse. ****p < 0.0001.

The gut microbiome impacts intestinal barrier function and can modulate colonic inflammation (51, 52). Because we observed enhancement of mucin gene expression after administration of the Qa-1^b^–agonistic peptide in the colitis model, we examined whether the Qa-1^b^ pathway has any impact on intestinal microbiota or metabolome that could affect the epithelial barrier even during steady state. We therefore collected fecal samples for 16S rRNA gene amplicon sequencing and untargeted metabolomics analysis from WT and Qa-1b^−/−^ mice of different ages and sex housed in our animal facility and at two different time points separated by 6 mo. We adopted this randomized sampling to avoid potential confounding influences, such as cage-associated effect, on the microbiota other than Qa-1b deficiency. Principal-component analysis (PCA) of the robust Aitchison distances based on the 16S sequencing data revealed that the microbiome profiles of WT and Qa-1b^−/−^ mice were significantly different (PERMANOVA *F* = 73.5, *p* < 0.001) (Fig. 7A). The genera *Prevotella* and *Akkermansia* appeared to drive the separation between the genotypes with lesser contributions by the genera *Bacteroides* and *Bacillus* along with an undetermined genus from the family Rikenellaceae. Additionally, α diversity, as measured by the Shannon index, was significantly higher in Qa-1b^−/−^ mice compared with WT (Mann-Whitney *U* = 3166, *p* = 3.8e 12, Fig. 7B). Further analysis of microbes that were differentially abundant between the groups were examined via Songbird (53), revealing that Qa-1b^−/−^ mice had higher proportions of the genera *Prevotella*, *Bacteroides*, and *Lactobacillus*, whereas WT mice were associated with higher proportions of *Akkermansia* and *Bacillus*. Differential abundance of *Akkermansia* was of particular interest because the genus is known to affect mucin production and regulate responses of the gut epithelium in mice and in humans (54–56). To confirm that abundance of *A. muciniphila* was different between WT and Qa-1b^−/−^ mice, we measured *Akkermansia* abundance by qPCR and found that the absolute abundance of *Akkermansia* in Qa-1b^−/−^ mice was significantly lower than in WT mice (Mann-Whitney *U* = 603, *p* = 4.6e 11) (Fig. 7C).

As a measure of the potential functional consequences of the microbial changes, we performed untargeted metabolomics on the same fecal samples used for 16S analysis. Similar to the microbiome, PCA of the β diversity based on robust Aitchison distances revealed that the metabolite profile of Qa-1b^−/−^ mice was significantly different from WT (PERMANOVA *F* = 14.5, *p* < 0.001) (Fig. 7D). Several of the metabolites most associated with Qa-1b^−/−^ mice are known, as are novel bile acids (57), such as cholic acid and hyocholic acid. Bile acid abundance and composition vary due to both host and microbial contributions (57), and bile acids may be conjugated in the liver, which affects their functions in fat absorption in the intestine. Qa-1^−/−^ mice had a higher ratio of unconjugated to conjugated primary bile acids compared with WT mice (Mann-Whitney *U* = 178, *p* = 3.6e−10) (Fig. 7E) based on Songbird analysis (53).

### In steady state, Qa-1b^−/−^ mice harbor a proinflammatory environment in colon and are accordingly more sensitive to DSS-induced colitis

**FIGURE 8.**
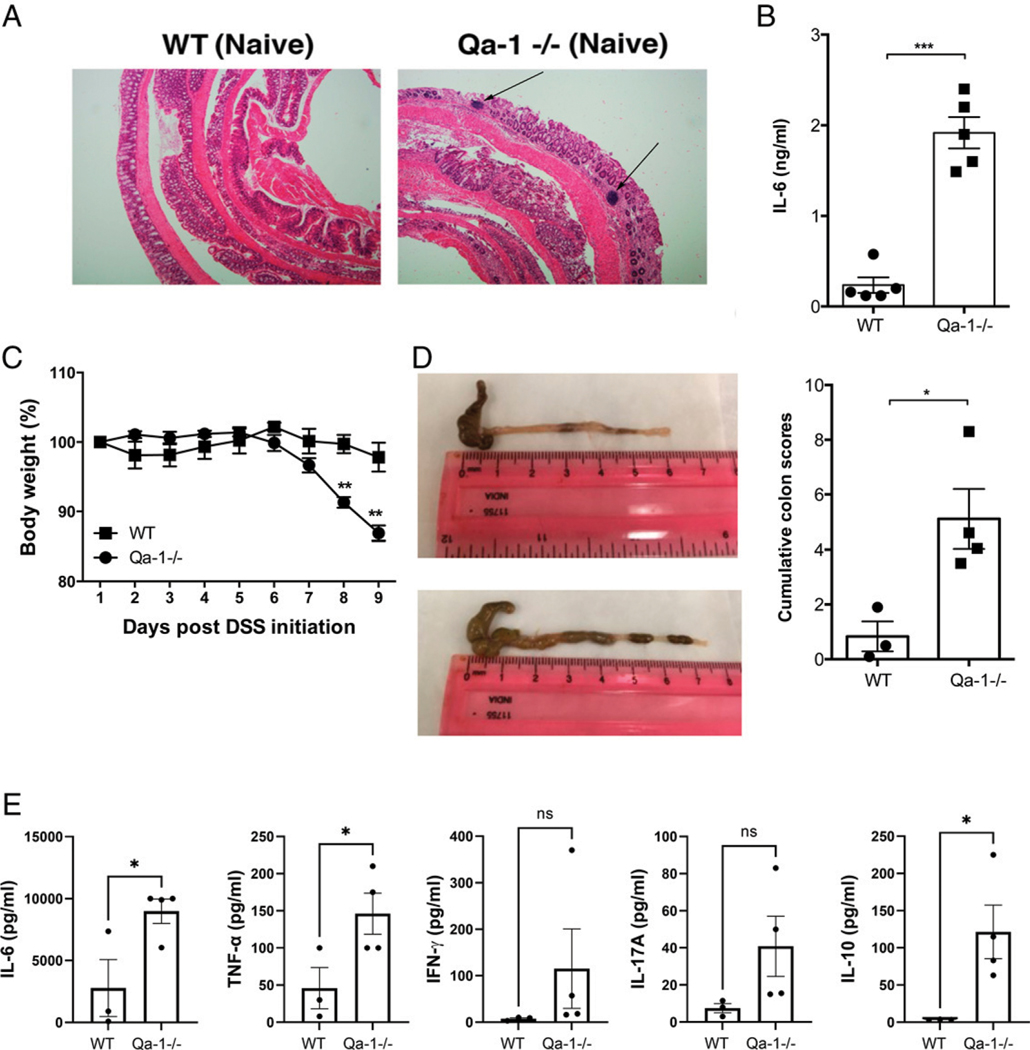
Spontaneous proinflammatory milieu of colonic tissue and enhanced sensitivity to DSS-induced colitis in Qa-1b^−/−^ mice (**A**) Representative images showing H&E staining of colon from WT (left panel) and Qa-1b^−/−^ (right panel) mice (*n* = 3–5). Arrows indicate accumulation of lymphocytes. Bar graph represents quantification of IL-6 in the supernatant of explant culture from naive mice by ELISA. (**B**) Comparative body weight loss between Qa-1b^−/−^ and WT counterparts using 1.5% DSS-mediated acute colitis model. (**C**) Representative photographs showing colons from WT or Qa-1b^−/−^ mice from acute DSS colitis model (1.5%). (**D**) Bar graph showing cumulative colitis scores from 1.5% acute DSS colitis model. Each dot represents one animal. Error bars represent mean ± SEM. (**E**) Bar graphs showing concentration (pg/ml) values from CBA kit analysis of cytokines (IL-6, TNF-α, IFN-γ, IL-17A, and IL-10) released in supernatant from colon explant culture. Colons were from WT or Qa-1b^−/−^ B6 mice from acute DSS colitis model (1.5%). Data are representative of two independent experiments. Each dot represents one animal. Error bars represent mean ± SEM. A Student’s *t* test was used to determine statistical significance. *p < 0.05, **p < 0.01, ***p < 0.001.

Next, we investigated whether the colonic tissues in steady state in Qa-1b^−/−^ mice harbor an enhanced proinflammatory milieu in the steady state. We found enhanced lymphocyte infiltrations into colonic tissue of Qa-1b^−/−^ in comparison with WT mice (Fig. 8A). Consistently, TCRαβ^+^CD8αβ^+^ adaptive T cells are significantly increased in steady state in the IEL in these mice (Supplemental Fig. 2D). Additionally, in the tissue explant culture, we also observed significantly higher levels of IL-6 in Qa-1b^−/−^ mice in comparison with WT mice (Fig. 8A). Because CD4 Tregs are important to control inflammation, we also investigated CD4^+^Foxp3^+^ Tregs to probe whether Qa-1b deficiency compromised colonic CD4^+^ Tregs. We did not find a significant reduction in colon IEL and colon LP fractions of CD4^+^Foxp3^+^ Tregs in Qa-1b^−/−^ mice in comparison with WT mice (Supplemental Fig. 2E).

We then investigated whether Qa-1b^−/−^ mice, because of this proinflammatory milieu, respond differentially in the induction of colitis. We chose a dose of DSS that does not precipitate properly into colitis in WT mice in our animal house, i.e., 1.5% DSS instead of the regular 2.5% DSS. When feeding age-matched mice with 1.5% DSS, we observed that whereas the WT mice did not respond with a change in body weight loss, Qa-1b^−/−^ counterparts suffered significant loss of body weight (Fig. 8B). Accordingly, Qa-1b^−/−^mice treated with DSS also developed enhanced gross and histopathological features of colitis (Fig. 8C, 8D). Explant culture of colon tissue demonstrated upward trends in proinflammatory cytokines from Qa-1b^−/−^ mice treated with low-dose DSS (Fig. 8E). Taken together, our data demonstrate a proinflammatory milieu in colon when the Qa-1b-based regulatory pathway is absent.

### IBD patients display a unique pattern of expression of HLA-E, an ortholog for the murine Qa-1^b^

**FIGURE 9.**
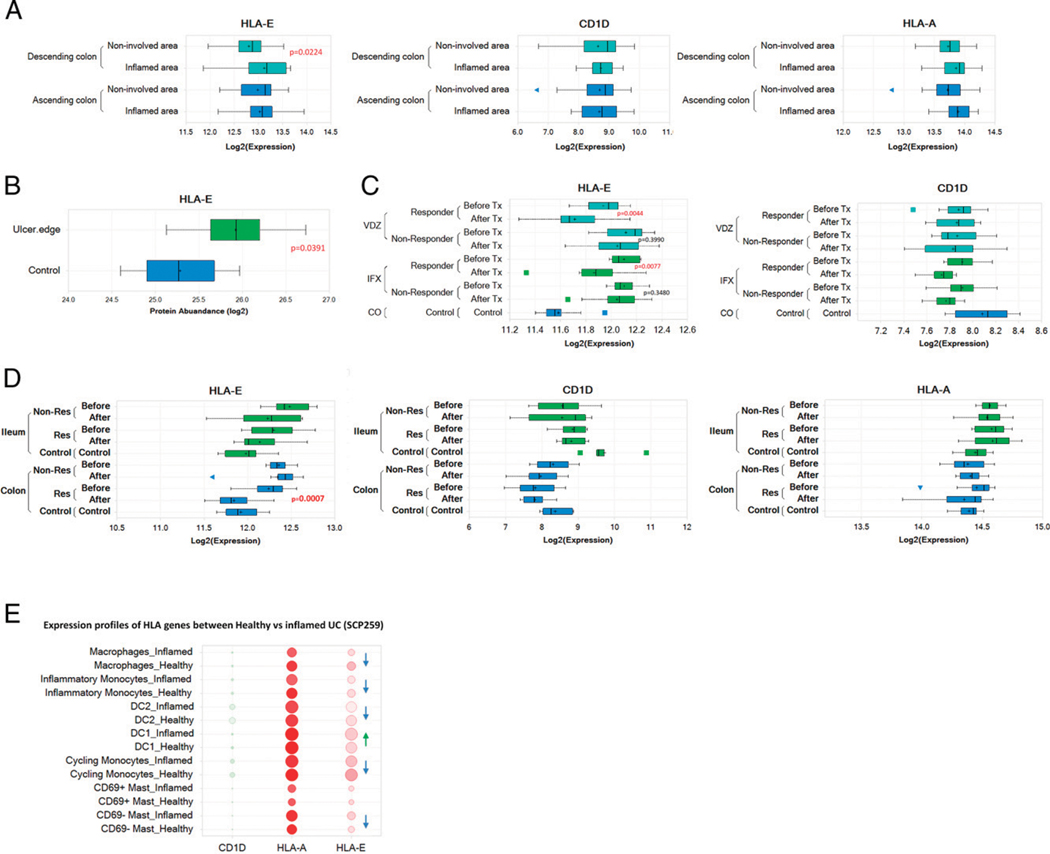
Enhanced HLA-E expression in inflamed tissue, with biologic non-response and in selected myeloid cell populations (**A**) Box plot showing different class I MHC molecules (HLA-E, CD1D, and HLA-A) expression levels in the groups of inflamed and non-involved regions from ascending colon (*n* = 48) and descending colon (*n* = 40) from CD patients (GSE100833). The *p* value was calculated from two-way ANOVA. (**B**) Box plot diagram showing the HLA-E protein expression levels in patients with colonic CD. Box plot showing HLA-E protein expression levels in the groups of ulcer edge (*n* = 8) and non-involved regions (*n* = 8) (PXD012284). (**C**) Box plot diagram showing the HLA-E and CD1D expression levels in patients with UC (GSE73661) in the groups of treatment response from vedolizumab (*n* = 18) and infliximab therapy (*n* = 23). The *p* value was calculated from two-way ANOVA. (**D**) Box plot showing HLA-E, CD1D, and HLA-A expression levels in the groups of infliximab treatment response in ileal (*n* = 18) and colonic CD (*n* = 19) (GSE16879). The *p* value was calculated from two-way ANOVA. (**E**) Dot plot diagram showing the gene expression levels in myeloid cells from UC patients (*n* = 18) and healthy individuals (*n* = 12) (SCP259). Dot size is proportional to prevalence of CD1D^+^/HLA-A^+^/HLA-E^+^ cells in each cell type. Dot color is proportional to gene average abundance in each cell type. Arrowhead indicates trend in increase of expression for HLA-E (in green for cDC1 as opposed to blue for other myeloid cell types).

Similar to the murine non-classical MHC molecules, humans also express a conserved class Ib MHC molecule, HLA-E, which shares several features with the murine counterpart (58, 59). Therefore, we determined whether HLA-E expression is altered in IBD patients and more specifically in any specific cell subset recently reported in IBD (5). We first compared differential gene expression of two class Ib MHC molecules (HLA-E and CD1d) and one class Ia MHC molecule (HLA-A) between non-inflamed and inflamed colon from descending and ascending colon in refractory Crohn’s disease (CD) using the CERTIFI cohort (GSE100833) (60). Human gene expression data were downloaded from the Gene Expression Omnibus database (60–62). Interestingly, in the descending colon, but not the ascending colon, we observed significantly enhanced gene expression of HLA-E, but not CD1d or HLA-A (Fig. 9A). The enhanced HLA-E gene expression was also supported by increased protein expression from a different colonic CD cohort (63), which included treatment-free samples and samples from patients treated with various medications (Fig. 9B). Human proteomics data were downloaded from the Proteomics Identification Database (63). Next, expression analysis of therapeutic response studies with Infliximab and vedolizumab allowed us to look into the induced restitution phase of the inflammatory disease in patients. In one study, colonic mucosal biopsies were obtained from ulcerative colitis (UC) patients treated with either vedolizumab or infliximab. Samples from before and after therapy along with outcome (response by histological healing to the therapeutic intervention or not) were compared with non-IBD controls (62). In another study, results for before and after the first infliximab treatment in ileal versus colonic CD were compared with those for non-IBD controls (61). In UC colon datasets and in colonic CD, but not in ileal CD, we observed decreased gene expression of HLA-E and not of CD1d and HLA-A (observed in CD) in therapeutic response and also not in non-responders (Fig. 9C, 9D). Next, on a cellular level, taking cue from our murine studies, mapping prominent myeloid cell subsets in UC obtained from a published scRNA-seq dataset (5), we observed overall enhanced expression of HLA genes similar to our observations as in Fig. 9A. Interestingly, only in cDC1, but not in other myeloid subsets, there was an enhancement of HLA-E expression by both gene average expression level and by abundance (Fig. 9E, Supplemental Table I). Thus, a similarity in the cell-specific expression profile of HLA-E in humans and Qa-1b in mice in anatomically similar inflamed tissues suggests potential involvement of this regulatory axis in IBD.

## DISCUSSION

In this study we show the dynamic nature of TCRαβ^+^CD8αα^+^ versus TCRαβ^+^CD8αβ^+^ T cell subsets that reside in the epithelial layer of colon in the steady state and during colitis. Notably, in the induction phase of the inflamed state, the frequency of TCRαβ^+^CD8αα^+^ T cells decrease with a significant increase in the frequency of the TCRαβ^+^CD8αβ^+^ T cells, and in resolution phase the reverse trends are observed. Expression of a non-classical class I MHC molecule, Qa-1b, which is known to be involved in regulatory CD8^+^ T cell-mediated immune regulation, is significantly upregulated in the Batf3 controlled cDC1s in inflamed epithelium. An agonistic peptide that stimulates Qa-1^b^-restricted regulatory TCRαβ^+^CD8αα^+^ T cells confers protection in the acute model of colonic inflammation in a Qa-1b- and Batf3-dependent manner, and enhances expression of known barrier-protective genes in distal colon. Consistent with a crucial role of the Qa-1^b^ regulatory axis, deficiency of Qa-1^b^ molecules in the steady state leads to a proinflammatory milieu in distal colonic tissue with a distinct microbiota, including a significant decrease in mucin-metabolizing bacteria and a significant alteration in the conjugated versus unconjugated primary bile acids. Expression of the human ortholog of Qa-1^b^, HLA-E, is overall enhanced with inflammation in colonic tissues from IBD patients and in biologic non-response subjects. Among myeloid cell subsets in humans, similar to our observations in mice, HLA-E expression in cDC1s also increases in comparison with all other myeloid cell subsets examined. Collectively, these studies identify a novel colonic immune regulatory mechanism involved in the maintenance of gut-barrier homeostasis.

CD8^+^ T cells with regulatory activity to counter proinflammatory T cells have been reported for a long time. Reports on CD8^+^ Treg phenotype have also been published (9, 10, 12, 14–19, 64, 65). Although both class Ia and class Ib MHC molecules have been implicated, a large number of studies have focused on regulatory CD8^+^ T cells restricted by the class Ib MHC molecule, Qa-1^b^ (9, 10, 12, 14–17, 64, 65). Recently, in both EAE and in patients with multiple sclerosis, classical class I MHC–restricted CD8^+^ Tregs have also been recently described (66). Several studies have also shown that CD8^+^ Tregs were either reactive to novel peptide epitopes or peptides derived from the TCR b-chain (12, 14, 15) and inhibited EAE by suppressing the anti-myelin oligodendrocyte glycoprotein encephalitogenic CD4^+^ T cells. We have recently identified a novel immunoregulatory TCRαβ^+^CD8αα^+^ T cell subset in both mice and humans with innate-like features that is dependent on the transcription factor PLZF capable of controlling both EAE and T cell-induced colitis (9). Consistently, glatiramer acetate-mediated induction of Qa-1 restricted lymph node-derived CD8^+^ T cells has been shown to also prevent DSS-induced colitis (67).

Our data indicate that in contrast to TCRαβ^+^CD8αβ^+^ T cells in colonic IEL or colonic LP CD8^+^ T cells, TCRαβ^+^ CD8αα^+^ T cells displaying a regulatory phenotype in steady-state CD44^+^CD122^lo^Ly49CIFH^+^ T cells mostly reside within the colonic IEL CD8αα^+^ T cells in comparison with CD8αβ^+^ T cells. Consistently, Helios^+^ Foxp3^−^CD8^+^ Tregs are also enriched in the colonic IEL CD8αα^+^ T cells in the steady state (data not shown). These data indicate that colonic IEL is a special repository of potential immunoregulatory TCRαβ^+^CD8αα^+^ T cells. Thus, TCRαβ^+^CD8αα^+^ T cells in colonic IEL are reduced in the inflammatory disease phase and are restored in the resolution phase, in sharp contrast to the adaptive CD8αβ^+^ T cells, a situation analogous to TYROBP^+/−^ IELs in human colon in UC (8). Importantly, in addition, note that these alterations in the ratio of CD8αα^+^ T versus CD8αβ^+^ T cells are restricted to the colonic IEL population and are not reflected in the colonic LP counterparts in mice, thus indicating epithelial layer damage and restoration in acute DSS colitis. Collectively, our data suggest that the TCRαβ^+^CD8αα^+^ T cells, or a subpopulation thereof, within colonic IEL support the integrity of the epithelial barrier from inflammatory assault by interacting with their immediate and prominent neighbors, that is, the epithelial cells or other immune cells within the epithelium.

Notably, during inflammation, Qa-1b molecules are significantly expressed on the cDC1 subset defined by the CD103^+^CD11b^−^ phenotype. Usually this subset resides in LP, but we observed its accumulation in the inflamed epithelial fraction. This is similar to another recent report where this subset of DCs was shown to communicate with epithelial cells in conferring protection in the colitis model (68). These DC subsets are known to be regulated by the transcription factor Batf3, and consequently the Qa-1b peptide-dependent regulation failed to protect Batf3^−/−^ mice. Loss of protection from colitis in Qa-1b^−/−^ mice further confirms an important role for the class Ib MHC-based regulatory axis. It is likely that in Qa-1b^−/−^ mice, either the Qa-1b-bound peptide is not presented to TCRαβ^+^CD8αα^+^ T cells and therefore these cells are not activated and thus unable to control colitis. Because most of the CD44^+^CD122^lo^Ly49CIFH^+^ CD8αα^+^ T cell population in colonic IEL is not lost in Qa-1b^−/−^ mice (data not shown), in the future we will investigate whether these cells are functionally or transcriptionally compromised in the absence of Qa-1b molecules. In summary, these data clearly demonstrate that the cDC1^−^CD8^+^ Treg regulatory axis, operating in the inflamed epithelium, nurtures the barrier in the time of distress or damage.

Importantly, investigating recently published scRNA-seq datasets from UC and healthy colon biopsy (5), we observed a small upward trend in HLA-E expression in cDC1s, but in contrast to all other myeloid cells studied. It is noteworthy that there is well-known lineage marker conservation between human and mouse cDC1s in the form of Clec9a/XCR1/BTLA/Necl2 positivity and CD14/Sirpα negativity (69, 70). Our data demonstrating the dependence of a Qa-1^b^ peptide-mediated protection on cDC1s suggest that it will be important to investigate whether a similar HLA-E peptide-dependent cDC1 immune regulatory axis is operational in humans. Interestingly, epithelial and stromal cells have high expression of HLA-E in human colonic datasets in UC [(8) and data not shown] and could play an important role in activating regulatory CD8^+^ T cells in human gut.

Our finding of the Qa-1b-dependent peptide-augmenting gene expression of the well-known barrier protective molecules, such as Klf4 and Muc2, provides a link of the immune system to the microbiome and non-immune compartment. Klf4 is a transcription factor with fundamental roles demonstrated in intestinal epithelial cells, including differentiation of colonic goblet cells (71, 72) and maintenance of cell morphology and polarity (73). Notably, epithelium-specific ablation of Klf4 results in enhanced pathology in the colitis-associated colorectal cancer model (74). In future studies we will further investigate role of the Qa-1b agonist peptide in goblet cell differentiation and mucus production in the context of colonic epithelium. Muc2^−/−^ mice elicit spontaneous colitis, affirming its overall protective role in gut inflammation (49). Mucus secretion is a property of the specialized epithelial cells called goblet cells, and indeed the CD103^+^ DCs have been shown to capture luminal Ags from Goblet cells and help in protection (75). However, because Muc2 has been shown to enhance tolerogenic DCs in gut (50), there may be the possibility for a feedback mechanism between Muc2 and tolerogenic DCs induced by the peptide. Interestingly, we observed enhanced Muc2 expression specifically in the distal colon, a region that was also used for our colon explant assays. In UC patients, pathology can be region specific. Thus, this immunoregulatory mechanism, if the biology holds true in humans, may be targeted for patients suffering from inflammation at distal or sigmoid colon. Important support data came from our in silico human studies, in which we observed that in colonic CD patients, HLA-E was augmented preferentially in descending colon as opposed to ascending colon. CD8^+^ Tregs have been recently demonstrated to be induced by commensal microbes in the context of protection from type 1 diabetes (76). We found that Qa-1b^−/−^ mice also had a deficiency in mucin-metabolizing microbiota *A. muciniphila*, whose members, like *A. muciniphila*, have been described as beneficial commensals in gut, and their potential in IBD therapeutics is being considered (77–79). Thus, this unique immune regulatory axis involves interactions among regulatory CD8^+^ T cells and mucin-metabolizing bacteria that can be exploited for therapeutics.

We also found a preponderance of primary bile acids in the absence of the regulatory axis in the stool of Qa-1b^−/−^ mice, reminiscent of a recent finding in IBD patient samples (80). Indeed, these enormous changes in microbiome and metabolome in the steady state was associated with a proinflammatory status in the colonic tissue in Qa-1b^−/−^ mice. Notably, the altered unconjugated/conjugated bile acid profile in these mice may suggest either altered liver function and/or reduced secondary modification by gut bacteria and needs further investigation.

Among the cytokines, IL-6, which is one of the prominent proinflammatory cytokines in murine colitis models and IBD patients (81, 82), was found to be significantly enhanced in Qa-1b^−/−^ mice without additional inflammatory stimulus. Whether the altered microbiome was the cause of the proinflammatory environment in the steady state in the absence of Qa-1^b^ needs to be further investigated. Because Qa-1b deficiency did not lead to a loss of CD4^+^Foxp3^+^ Tregs in colon, the functional loss of the TCRαβ^+^CD8αα^+^ Treg-centered regulatory axis accounts for the control of immunoregulation in the colon, thereby suggesting their important role in the maintenance of epithelial barrier function. It is likely that in the steady state, interactions exist between gut microbiota and Qa-1^b^-expressing cDC1s that may cross-present microbial peptides to TCRαβ^+^CD8αα^+^ T cells to maintain membrane integrity. Importantly, these data indicate that both CD4 Tregs as well as CD8^+^ Tregs are important for the maintenance of immune homeostasis in colon. Our findings also are important in that by defining agonistic peptides for both class Ia and class Ib MHC-restricted CD8^+^ Tregs, we provide a unique biologics or synthetic chemistry opportunity for potential novel therapeutics for IBD. With literature being enhanced with scRNA-seq and other deep-sequencing studies from IBD patients, human-relevant CD8^+^ Treg mechanisms should be revealed in the near future.

## Supplementary Material

supplemental

## Abbreviations used in this article:

B6: C57BL/6
CBA: cytometric bead array
CD: Crohn’s disease
cDC1: type 1 conventional dendritic cell
DSS: dextran sulfate sodium
EAE: experimental autoimmune encephalomyelitis
IBD: inflammatory bowel disease
IEL: intestinal epithelial layer
Klf4: Krüppel-like factor 4
LP: lamina propria
MLN: mesenteric lymph node
PCA: principal-component analysis
pDC: plasmacytoid DC
PERMANOVA: permutational ANOVA
qPCR: quantitative PCR
scRNA-seq: single-cell RNA sequencing
Treg: regulatory T cell
UC: ulcerative colitis
WT: wild-type
