# Supplementary material for: Class Ib MHC–Mediated Immune Interactions Play a Critical Role in Maintaining Mucosal Homeostasis in the Mammalian Large Intestine
Source: Immunohorizons. Author manuscript; Available in PMC 2023 Mar 20. (PMC10026853; doi:10.4049/immunohorizons.2100090)
Supplement: supplemental [file NIHMS1868228-supplement-supplemental.pdf]

Supplementary Figure 1

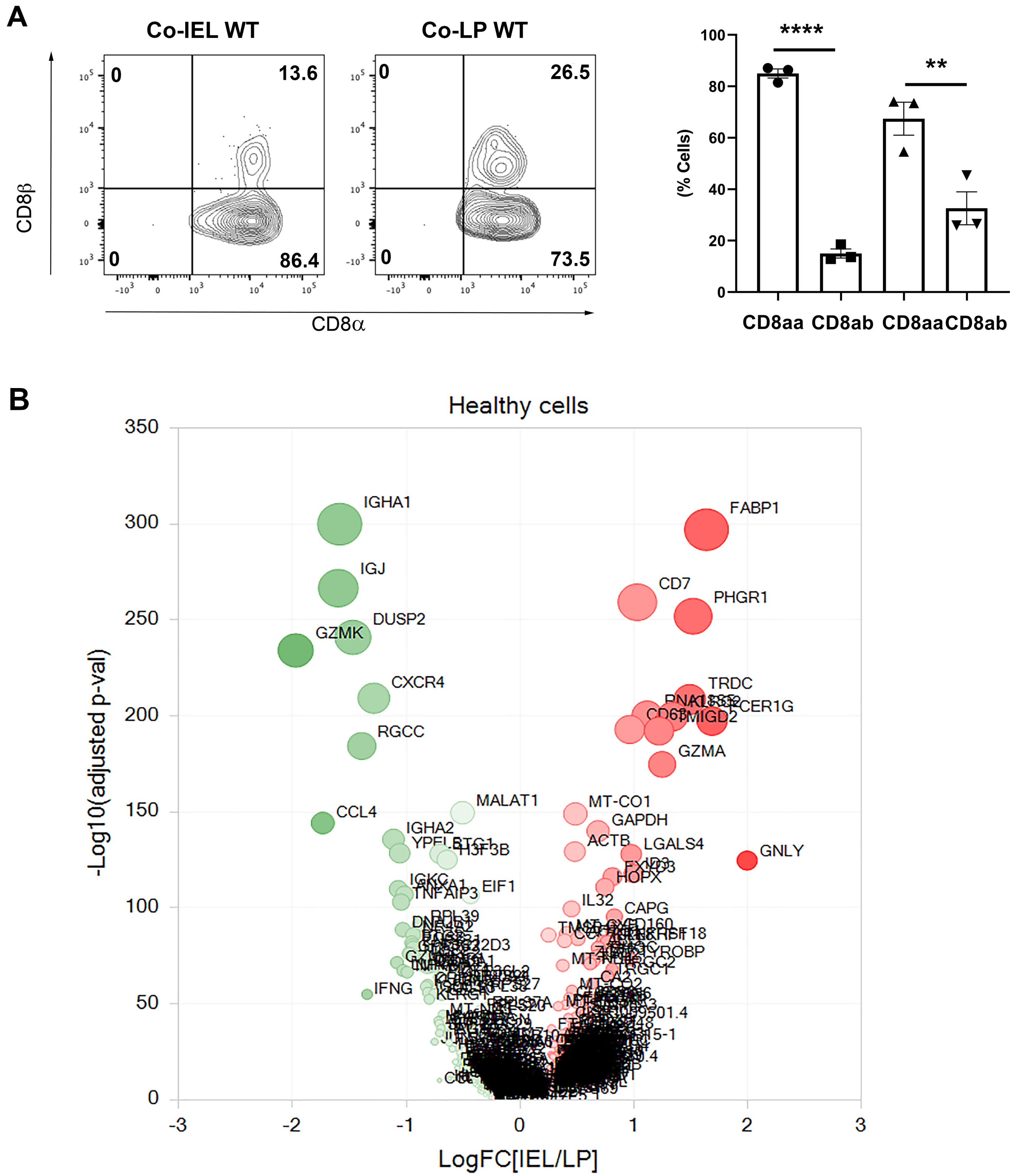

Supplementary fig 1. Steady state features of IEL CD8+ T cells in mammalian colon. A) Single cells were isolated from colon (IEL and LP) of WT B6 mice and analyzed by flow cytometry. The enrichment of CD8aa T cell compartment (gated as TCRb+CD4+Helios+CD8a+FOXP3-) in Helios+ CD8aa+ T cells in comparison to that in the CD8ab compartment, in both Colonic IEL and LP is shown in representative Flow plots (3rd quadrant over 2nd quadrant) and bar graph. Each dot in the bar graph represents one mouse. Error bars represent mean ± SEM. One way Anova was used to determine statistical significance. **** p <0.0001; ** p<0.01. B) Differential expression of CD8+ IEL vs CD8+ LP in healthy donors. Volcano plots comparing Log2 (fold change) of gene expression in CD8+ IEL vs CD8+ LP cells from healthy donors (n=12). Published datasets from Smilie, CS et al. (2019) were re-analyzed in silico (see Material and Methods for details). Differential gene analysis was performed using data from 12 healthy donor samples, as mentioned in the original article. Significantly up-regulated genes shown in red, significantly down-regulated genes shown in green (adjusted p<0.05).

Downloaded from http://journals.aai.org/immunohorizons/article-supplement/234081/pdf/ih_2100090_supplemental_1/ by Univ of California-San Diego Serials/Biomed Lib 0699 user on 10 March 2023

Supplementary Figure 2

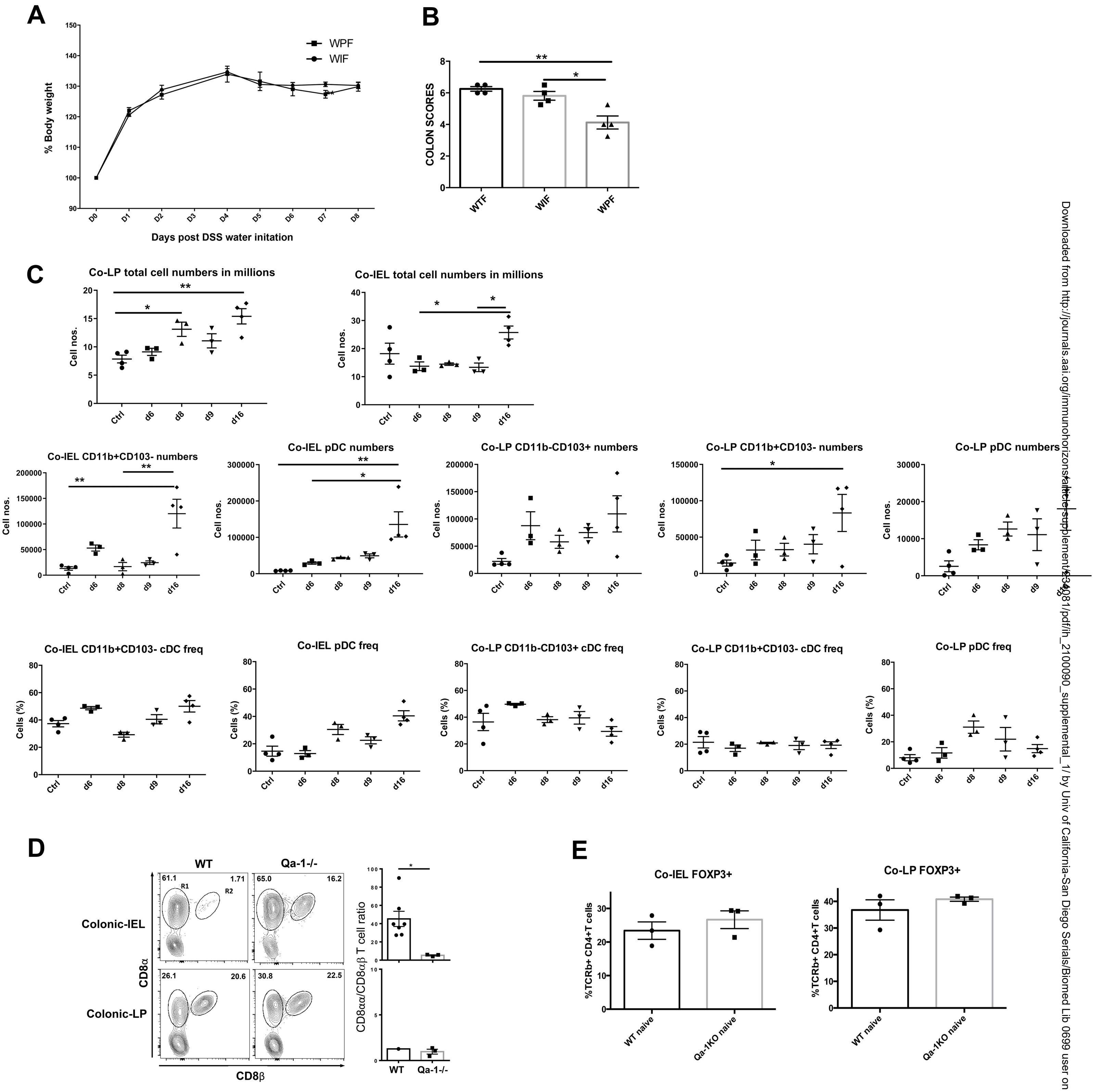

Supplementary fig 2. Selected parameters in DSS colitis model and in steady state. A) IFA/buffer alone rectifies body weight loss but does not protect colon from acute DSS colitis.WT B6 (n = 3-7 per group) mice were fed 2.5% DSS (or not for vehicle controls) in water for 7 days and then switched to regular water. (A-B) Mice were pre-treated with Qa-1b-binding peptide or not before the start of the DSS cycle. Representative data from two independent experiments are shown. In WT B6 mice IFA/buffer alone (WIF) leads to body weight difference which is not modulated by the peptide (WPF) B) IFA/buffer (WIF) does not have effect on colon compared to WT B6 mice treated with DSS (WTF) and the observed effect is peptide-mediated (WPF). Error bars represent mean ± SEM. Non-parametric (Mann Whitney) test was used to determine statistical significance. C) Mice were sacrificed at indicated time points along the colitis model. Frequency of cDC2 (% of CD11b+CD103- events in live CD45+MHCII+CD3-CD19-CD11c+ cells) and pDCs (% of SH+PDCA-1+ events in live CD45+CD3-B220+CD11c+ cells) in Co-IEL and Co-LP and corresponding absolute numbers in DSS colitis. Untreated WT and Qa-1b-/- mice were investigated for the presence of CD8+T cells (D) and CD4+FOX P3+ Tregs in colonic IEL (E, left panel) and LP (E, right panel). Frequency of CD4+FOXP3+ T cells do not change in colon of Qa-1b-/- mice, though a skew in CD8aa/CD8ab T cells are observed in IEL. Representative from two independent experiments. Each dot represents one mouse.

Downloaded from http://journals.aai.org/immunohorizons/article-supplement/234081/pdf/ih_2100090_supplemental_1/ by Univ of California-San Diego Serials/Biomed Lib 0699 user on 10 March 2023

Supplementary Figure 3

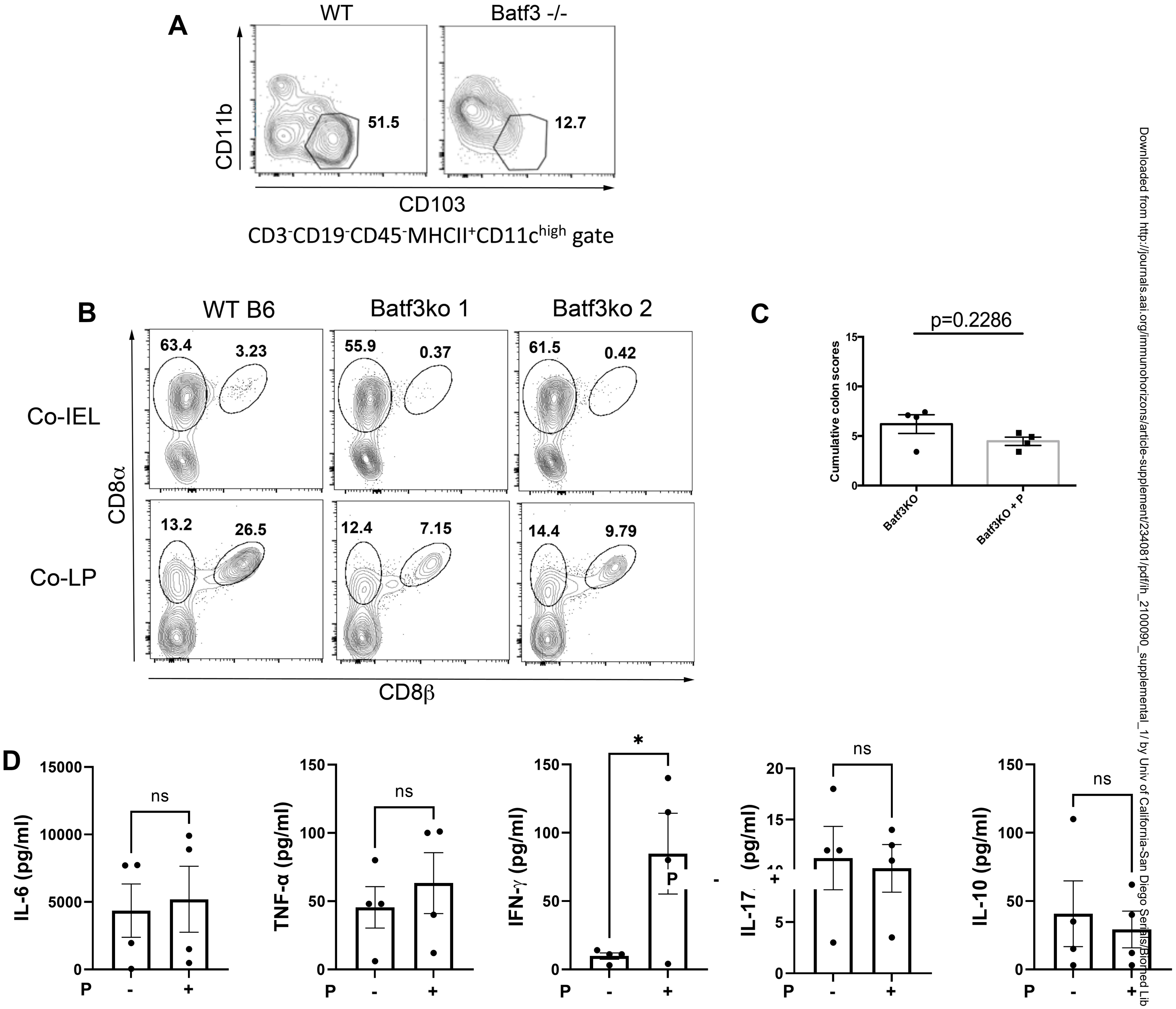

Supplementary fig 3. Batf3 dependence of cDC1 and CD8 T cells (A,B) in steady state colon tissue. Untreated WT and Batf3-/- mice were investigated for the presence of cDC1 (CD103+CD11b- cDCs) in colonic LP (A). Representative of 2 experiments is shown. (B) TCRαβ+CD8αα+ and TCRαβ+CD8αβ+ T cells in colonic IEL (top panel) and colonic LP (bottom panel). (C-D) Lack of protection by peptide at higher dose of DSS. Batf3-/- mice were pre-administered with Qa-1b-binding peptide or not and then treated with 3.5% DSS in drinking water for 7 days and then switched to regular water.

Downloaded from http://journals.aai.org/immunohorizons/article-supplement/234081/pdf/ih_2100090_supplemental_1/ by Univ of California-San Diego Serials/Biomed Lib 0699 user on 10 March 2023

Supplementary Table

Cell	Gene	Healthy expression	Inflamed expression	Healthy prevalence (%)	Inflamed prevalence (%)
CD69- Mast	CD1D	0.00	0.00	0.00	0.00
CD69- Mast	HLA-A	15.45	15.23	73.33	83.33
CD69- Mast	HLA-B	10.79	26.77	65.00	90.00
CD69- Mast	HLA-C	16.43	19.59	86.67	86.67
CD69- Mast	HLA-E	4.52	5.24	45.00	60.00
CD69+ Mast	CD1D	0.01	0.00	0.11	0.08
CD69+ Mast	HLA-A	12.03	11.68	53.44	58.86
CD69+ Mast	HLA-B	14.35	16.47	57.31	67.00
CD69+ Mast	HLA-C	11.18	10.54	49.89	56.93
CD69+ Mast	HLA-E	4.90	4.60	34.62	36.43
Cycling Monocytes	CD1D	0.62	0.48	32.98	25.64
Cycling Monocytes	HLA-A	16.98	14.68	93.72	94.87
Cycling Monocytes	HLA-B	24.78	28.78	95.81	96.15
Cycling Monocytes	HLA-C	19.09	17.19	96.34	94.87
Cycling Monocytes	HLA-E	7.74	6.23	92.15	84.62
DC1	CD1D	0.16	0.15	12.69	8.28
DC1	HLA-A	26.02	19.11	97.76	98.73
DC1	HLA-B	45.04	40.60	99.25	100.00
DC1	HLA-C	27.27	21.53	98.51	98.09
DC1	HLA-E	5.06	5.58	83.58	92.99
DC2	CD1D	1.45	1.08	43.44	37.64
DC2	HLA-A	14.02	13.20	92.83	95.18
DC2	HLA-B	25.09	19.74	97.29	98.10
DC2	HLA-C	15.02	12.58	96.48	95.44
DC2	HLA-E	4.61	3.69	78.76	79.59
Inflammatory Monocytes	CD1D	0.51	0.53	12.20	11.70
Inflammatory Monocytes	HLA-A	15.63	11.59	80.49	81.57
Inflammatory Monocytes	HLA-B	24.86	17.45	95.12	87.64
Inflammatory Monocytes	HLA-C	16.63	11.88	87.80	80.24
Inflammatory Monocytes	HLA-E	4.74	4.24	59.35	55.41
Macrophages	CD1D	0.27	0.33	7.64	5.93
Macrophages	HLA-A	14.43	11.75	77.27	69.62
Macrophages	HLA-B	23.37	17.29	85.13	77.45
Macrophages	HLA-C	12.98	10.06	75.29	62.36
Macrophages	HLA-E	6.47	4.71	63.96	47.11

Supplementary table 1. Expression profiles of HLA genes between Healthy vs inflamed UC using in silico analysis of published scRNAseq datasets, Smilie, CS et al. (2019) (SCP259). Gene expression levels in myeloid cells from Ulcerative colitis patients (n=18) and healthy individuals (n=12) (SCP259). Arrowhead indicates trend in increase of expression for HLA-E

Downloaded from http://journals.aai.org/immunohorizons/article-supplement/234081/pdf/ih_2100090_supplemental_1/ by Univ of California-San Diego Serials/Biomed Lib 0699 user on 10 March 2023
